# Brahma-related gene 1 acts as a profibrotic mediator and targeting it by micheliolide ameliorates peritoneal fibrosis

**DOI:** 10.1186/s12967-023-04469-w

**Published:** 2023-09-19

**Authors:** Shuting Li, Congwei Luo, Sijia Chen, Yiyi Zhuang, Yue Ji, Yiqun Zeng, Yao Zeng, Xiaoyang He, Jing Xiao, Huizhen Wang, Xiaowen Chen, Haibo Long, Fenfen Peng

**Affiliations:** 1 Department of Nephrology, Zhujiang Hospital, Southern Medical University, Guangzhou, 510280 China; 2 Department of Nephrology and Rheumatology, The First Hospital of Changsha, Changsha, China

**Keywords:** Peritoneal fibrosis, BRG1, Micheliolide, TGF-β1, Peritoneal dialysis

## Abstract

**Background:**

Progressive peritoneal fibrosis is a worldwide public health concern impacting patients undergoing peritoneal dialysis (PD), yet there is no effective treatment. Our previous study revealed that a novel compound, micheliolide (MCL) inhibited peritoneal fibrosis in mice. However, its mechanism remains unclear. Brahma-related gene 1 (BRG1) is a key contributor to organ fibrosis, but its potential function in PD-related peritoneal fibrosis and the relationship between MCL and BRG1 remain unknown.

**Methods:**

The effects of MCL on BRG1-induced fibrotic responses and TGF-β1-Smads pathway were examined in a mouse PD model and in vitro peritoneal mesothelial cells. To investigate the targeting mechanism of MCL on BRG1, coimmunoprecipitation, MCL-biotin pulldown, molecular docking and cellular thermal shift assay were performed.

**Results:**

BRG1 was markedly elevated in a mouse PD model and in peritoneal mesothelial cells cultured in TGF-β1 or PD fluid condition. BRG1 overexpression in vitro augmented fibrotic responses and promoted TGF-β1-increased-phosphorylation of Smad2 and Smad3. Meanwhile, knockdown of BRG1 diminished TGF-β1-induced fibrotic responses and blocked TGF-β1-Smad2/3 pathway. MCL ameliorated BRG1 overexpression-induced peritoneal fibrosis and impeded TGF-β1-Smad2/3 signaling pathway both in a mouse PD model and in vitro. Mechanically, MCL impeded BRG1 from recognizing and attaching to histone H3 lysine 14 acetylation by binding to the asparagine (N1540) of BRG1, in thus restraining fibrotic responses and TGF-β1-Smad2/3 signaling pathway. After the mutation of N1540 to alanine (N1540A), MCL was unable to bind to BRG1 and thus, unsuccessful in suppressing BRG1-induced fibrotic responses and TGF-β1-Smad2/3 signaling pathway.

**Conclusion:**

Our research indicates that BRG1 may be a crucial mediator in peritoneal fibrosis and MCL targeting N1540 residue of BRG1 may be a novel therapeutic strategy to combat PD-related peritoneal fibrosis.

**Supplementary Information:**

The online version contains supplementary material available at 10.1186/s12967-023-04469-w.

## Introduction

Peritoneal dialysis (PD) comprises 9% of all kidney replacement therapy and 11% of all dialysis worldwide. According to the 2018 International Society of Nephrology Global Kidney Health Atlas, the median global prevalence of PD was 38.1 per million population [1, 2]. Furthermore, over 50% of PD patients reside in four countries (China, the United States, Mexico and Thailand) [2, 3]. However, Even if PD therapy achieves a health-related quality and outcome equivalent to hemodialysis [4, 5], long-term exposure to bioincompatible glucose-containing PD fluids trigger inflammatory, neoangiogenic, and profibrotic processes, culminating to peritoneal fibrosis, eventually resulting in discontinuation of PD therapy [6]. Novel therapies to prevent and alleviate the progression of PD-related peritoneal fibrosis are urgently needed.

During the development of peritoneal fibrosis, a particular form of cell transition in peritoneal mesothelial cells called mesothelial-to-mesenchymal transition (MMT) has been devoted considerable attention [7, 8]. Mesothelial cells undergoing MMT manifest in the expression of mesenchymal markers such as α-smooth muscle actin (α-SMA) and a lack of epithelial features, leading to a buildup of extracellular matrix (ECM) [9, 10]. Transforming growth factor-β 1 (TGF-β1) is a principal mediator of MMT [7, 11]. Upon its release, TGF-β1 binds to TGF-βRII, which then phosphorylates TGF-βRI, leading to the phosphorylation of Smad2 and/or Smad3 and their formation of a heterotrimeric complex with Smad4, which is then translocated into the nucleus. This complex eventually regulates pro-fibrotic gene overexpression and contributes to various types of tissue fibrosis such as peritoneal fibrosis [8–10].

Brahma-related gene 1 (BRG1), also known as SMARCA4, is the central catalytic ATPase of the switch/sucrose nonfermentable chromatin remodeling complex, which functions in mobilizing nucleosomes and higher-order chromosome dynamics to regulate gene transcription [12]. Evidences have been presented to suggest that BRG1 is a key contributor to fibrosis in the liver [13], heart [14], lung [15] as well as kidney [16, 17]. Our previous research revealed that BRG1 increases the expression of TGF-β1 in tubular epithelial cells [16], implying that BRG1 may be involved in TGF-β1-Smads signal cascade. Nevertheless, the potential function of BRG1 in PD-related peritoneal fibrosis and its underlying mechanism involving TGF-β1-Smads pathway are still largely unknown.

Previously we found that parthenolide (PTL), a sesquiterpene lactone originally extracted from the shoots of the plant Feverfew (Tanacetum balsamita) [18, 19] alleviated PD-associated peritoneal fibrosis by suppressing the TGF-β1-Smads pathway [20, 21]. Nevertheless, the clinical application of PTL in peritoneal fibrosis remains hindered owing to some disadvantages such as low aqueous solubility, weak oral bioavailability, and relative instability under chemical and physiological conditions [22]. As a result, to discover an alternative strategy to maximize the therapeutic value of PTL, micheliolide(MCL) was derived from PTL with improved water solubility, enhanced plasma stability (half-lives of MCL and PTL in mouse plasma, 2.64 vs 0.36) and decreased toxicity [23, 24]. Dimethylaminomicheliolide (DMAMCL), the pro-drug of MCL, has a high degree of bioavailability when administered orally. DMAMCL can be slowly and continuously converted to MCL for up to 8 h, and it can maintain the effective concentration of MCL in plasma and pass through the blood–brain barrier [25]. Moreover, we previously reported that DMAMCL ameliorates renal fibrosis [26] and peritoneal fibrosis [27]. Nevertheless, exact mechanism of MCL in peritoneal fibrosis remains to be clarified.

In this study, an in vivo PD mouse model and an in vitro MMT model in peritoneal mesothelial cells were established to investigate the potential role of BRG1 in PD-related peritoneal fibrosis and TGF-β1-Smad2/3 pathway. We also examined the role of MCL in BRG1-induced peritoneal fibrosis and its underlying mechanism. Our findings suggest that BRG1 may be a crucial mediator in peritoneal fibrosis and MCL targeting BRG1 may be a novel therapeutic strategy to treat PD-related peritoneal fibrosis.

## Materials and methods

### Mouse model and sample collection

Eight-to-ten-week-old male C57BL/6J mice were obtained from Southern Medical University Animal Center (Guangzhou, China). The animal protocol was reviewed and approved by the Animal Experimental Ethics Committee at Southern Medical University (Approval No. LAEC-2020-166). A mouse model of peritoneal fibrosis was established by daily intraperitoneal injection of 3 ml of standard PD fluid composed of 4.25% glucose(Baxter HealthCare, Deerfield, IL) for 4 weeks [27, 28]. To investigate the role of BRG1, in vivo overexpression of BRG1 in mice was performed by ultrasound-microbubble-plasmid containing mouse BRG1 expression plasmid (pBRG1) [16] or empty plasmid(Vector) treatment on days 0 and 14 over 28 days [28]. To investigate the role of DMAMCL in BRG1 overexpression-induced peritoneal fibrosis, five groups of mice were treated as follows: (1) the control group received daily intraperitoneal injection of 3 ml saline and ultrasound-mediated vector plasmid gene transfer treatment and daily intragastric administration of saline; (2) mice in the PD group received daily intraperitoneal injection of 3 ml PD fluid and ultrasound-mediated vector plasmid gene transfer treatment and daily intragastric administration of saline; (3) mice received PD fluid and ultrasound-mediated BRG1 expression plasmid gene transfer treatment and daily intragastric administration of saline; (4) mice received PD fluid and ultrasound-mediated vector plasmid gene transfer treatment and daily intragastric administration of DMAMCL (25 mg/kg); and (5) mice received PD fluid and ultrasound-mediated BRG1 expression plasmid gene transfer treatment and daily intragastric administration of DMAMCL (25 mg/kg).The detailed experimental design was shown in Fig. 5a.After the 28-day PD fluid treatment, the mice were sacrificed. Parietal and visceral peritoneal tissues were collected for further analysis.

### Histology and immunohistochemical staining of peritoneal samples

The thickness of the submesothelial layer was determined on 4-um paraffin-embedded tissue sections stained with Masson’s trichrome staining according to a routine procedure. The thickness of the submesothelial tissue was calculated by measuring the average distance from the superficial mesothelial cell layer to the muscle from 15 independent measurements for each animal. Immunohistochemical staining was conducted as previously described [27]. Paraffin sections (4-um thick) were subjected to deparaffinization in xylene and rehydrated in a series of decreasing alcohol concentrations. Subsequently, the sections were exposed to sodium citrate antigen retrieval solution at a sub-boiling temperature for 8 min in a microwave oven to restore the antigen. To inhibit the endogenous peroxidase of the samples, 3% H2O2 was applied for 15 min in a dark environment. Following this, the sections were incubated in PBS containing 3% BSA for 60 min at room temperature. After overnight incubation with primary antibodies at 4 °C, the samples were treated with secondary antibody for 30 min at room temperature. Finally, the tissue sections were exposed to diaminobenzidine (DAB) for 3 min at room temperature. Antibodies used were described in Additional file 1: Table S1.

### Primary RPMCs, immortalized human peritoneal cells HMrSV5 culture and treatment

Two different cell types were analyzed: primary rat peritoneal mesothelial cells (RPMCs) and immortalized human peritoneal cells (HMrSV5 cells). Primary RPMCs were derived from trypsin/ ethylenediaminetetraacetic acid digestion method from peritoneal tissue obtained from male SD rats (120 g–150 g). After centrifuged and washed, cells were cultured in DMEM/F-12 medium (Gibco)) containing 10% fetal bovine serum(Gibco), 1% antibiotics (100 units/mL penicillin and 100 mg/mL streptomycin,Gibco) and 1% Insulin-Transferrin-Selenium (Gibco) at 37 °C in 5% CO2. HMrSV5 cells (kindly provided by Professor Xueqing Yu, Sun Yat-Sen University,Guangzhou, China) were cultured in DMEM/F12 with 10% fetal bovine serum and 1% antibiotics at 37 °C in 5% CO2. For TGF-β1 treatment, cells were grown to ~ 70% confluence and starved with serum free DMEM/F-12 medium for 12 h and then treated with 5 ng/ml recombinant human TGF-β1 (R&D Systems, Minneapolis, MN)) for 48 h. For PD fluid treatment, serum-starved cells were cultured in conventional PD fluid (Baxter HealthCare, Deerfield, IL) for 48 h with different concentrations. Cells incubated in medium without stimuli or with medium diluted 1:2 with sterile saline served as a control for TGF-β1–stimulated and PD fluid-stimulated cells, respectively. In some experiments, cells were treated with TGF-β1 and cotreated with BRG1 inhibitor PFI-3 (2 μM, Selleck Chemicals), MCL (1.25 μM, 2.5 μM, 5 μM) in a serum-free medium for 48 h.

### Transfection of siRNA and plasmid

Cells were transiently transfected with BRG1-specific small interfering RNA (siRNA) (designed and synthesized by GenePharma, Shanghai, China) or siNC using lipofectamine 2000 reagent (Invitrogen, MA, U.S.A.). The siNC was used as  a negative control. Cells were transfected with pEZ-M14-FLAG plasmid containing full length BRG1, plasmid containing BRG1 mutant(asparagine 1540 was mutated into alanine,N1540A), BRG1 deletion mutant expression plasmids [N-terminal domain (1–765), C-terminal domain(1301–1647) or ATPase domain (766–1300)], plasmid containing H3 lysine 14 full length(H3K14WT) and two mutant plasmids (H3K14Q and H3K14R with lysine 14 mutated to glutamine and arginine, respectively) (all from GeneCopoeia) using Lipofectamine 3000(Invitrogen). The sequence of siBRG1 were shown in Additional file 1: Table S2.

### Western blot analysis

Total protein sample from tissues or cells were extracted with RIPA lysis buffer (KeyGen, Nanjing, China) according to the routine procedure. Cytoplasmic and nuclear proteins from RPMCs were separated by a commercial protein separation kit (P0028, Beyotime, China) according to the manufacturer’s protocol. Antibodies used were listed in Additional file 1: Table S1.

### Immunofluorescence

Cells cultured on coverslips were fixed with 4% formaldehyde followed by permeabilization with 0.1% Triton X-100. And then these slides were blocked with 5% goat serum for 1 h at room temperature. Subsequently, they were incubated with primary antibodies. After washing, slides were incubated with fluorescent-labeled secondary antibodies for 1 h at room temperature, and then nuclei were stained with 4′,6-diamidino-2-phenylindole (DAPI) (BestBio, Shanghai, China). Images were taken with confocal microscope (Nikon, Tokyo, Japan). Antibodies used were listed in Additional file 1: Table S1.

### RNA extraction and quantitative real-time-PCR

Total RNAs were isolated with RNAiso reagent (Agbio, China) and converted into cDNA using a Reverse Transcription System kit according to the instructions of the manufacturer (Agbio). And then the amplified product was used in Quantitative real-time-PCR ABI PRISM 7000 Sequence Detection System (Applied Biosystems). The sequences of the primers were presented in Additional file 1: Table S3.

### Coimmunoprecipitation

Cells were lysed on ice with NP-40 buffer for 20 min and then were centrifuged at 12,000 ×*g* for 15 min at 4  °C. The supernatants were collected and incubated with primary antibody and rocked overnight at 4  °C. Then, protein A/G (sc2003, Santa Cruz Biotechnology) was added and cells were incubated at 4  °C on a rocker platform for 4 h. The immunocomplexes were washed for three times and mixed with the loading buffer for degeneration at 100  °C for 10 min. The bound proteins were subjected to immunoblotting analysis. Antibodies used were described in Additional file 1: Table S1.

### Pull-down of biotin-MCL bound proteins

Whole-cell lysates were collected and equally divided into two parts. The one was incubated with unlabeled MCL (200 μM; negative control), and the other was incubated with biotin-MCL (200 μM), as described previously [29]. After incubation overnight at 4  °C, prewashed 30 µL streptavidin beads (Thermo Fisher Scientific, Waltham, MA, USA)were added to each samples and incubated for another 4 h at 4  °C. Then the beads were washed five times with PBS and the bead-bound proteins were eluted, followed by western blotting analysis. Antibodies used were described in Additional file 1: Table S1.

### Cellular thermal shift assay

HMrSV5 cells were treated with MCL (5 μM) or DMSO for 2 h, then lysed using lysis buffer supplemented with pierce protease and phosphatase inhibitor. Subsequently, after centrifugation, the supernatant was divided into twelve aliquots and heated with various temperature (56, 58, 60, 62, 64, 66, 68, 70, 72, 74, 76 and 78  °C) via PCR instrument respectively. After cooling to room temperature, soluble proteins were collected by centrifugation at 12,000*g* for 20 min at 4  °C and then subjected to western blotting.

### Molecular docking

Molecular docking was adopted to understand the potential interactions between MCL and BRG1. Discovery Studio 2017 R2 (BIOVIA Software, Inc., San Diego, CA, U.S.A.) software was employed in the present study. The structure of MCL was optimized by Minimize protocol. The X-ray crystal structure of BRG1 was downloaded from the RCSB Protein Data Bank. CDOCKER protocol was applied to run molecular docking. Other parameters were set as default.

### Statistical analyses

Data were expressed as mean ± SEM. Statistical analyses of the data were performed using SPSS 22. 0 (SPSS Inc, Chicago, IL). Independent-Student’s t test was employed for comparisons between two groups. One-way ANOVA followed by Least significant difference (LSD) test or Dunnett’s T3 procedure for multiple comparison was used for groups of three or more. Statistical significance was defined as P < 0. 05.

## Results

### BRG1 is elevated in a mouse PD model and in peritoneal mesothelial cells exposed to TGF-β1 or PD fluid

**Fig. 1:**
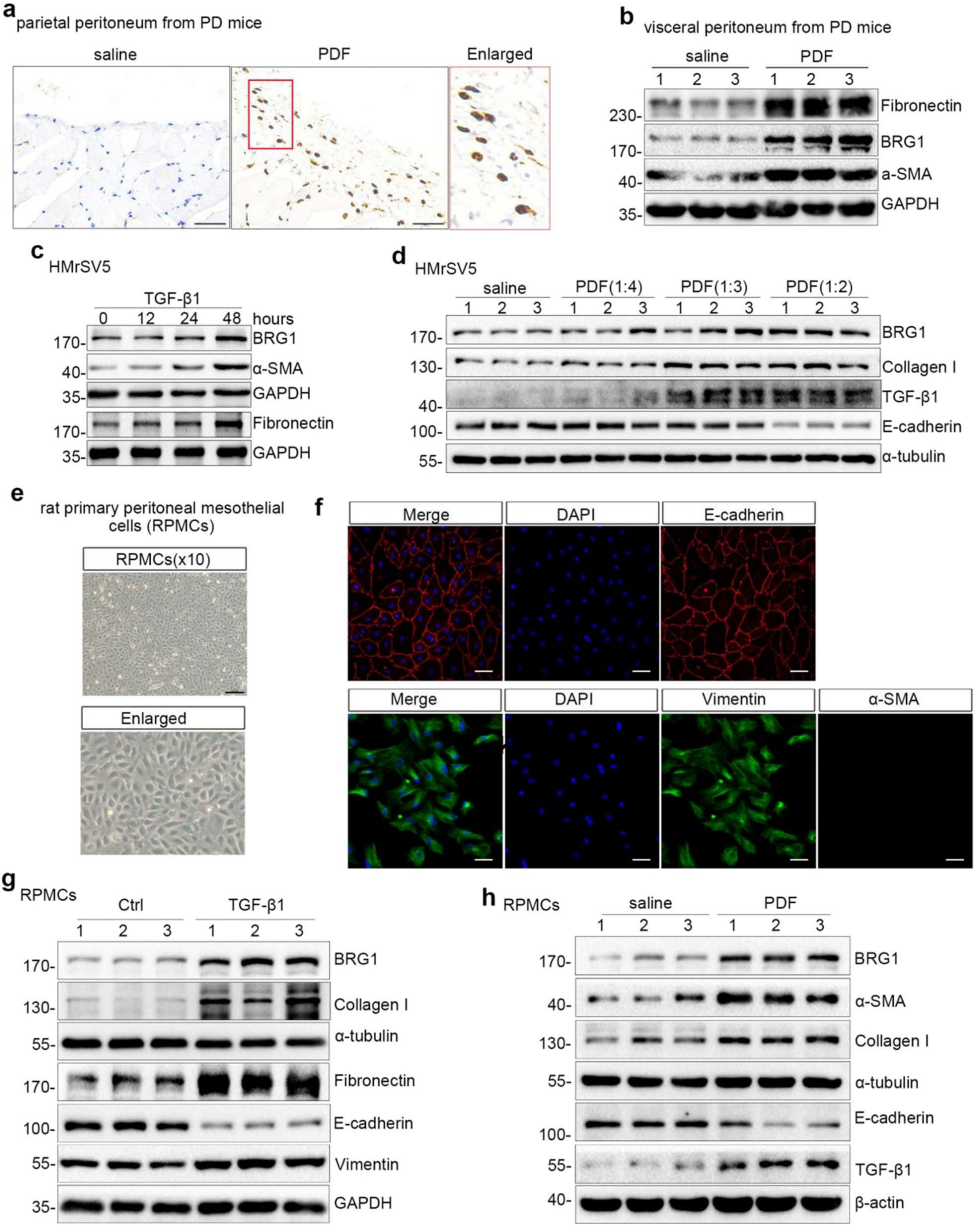
BRG1 is increased in PD mice and in TGF-β1-induced or PD fluid-treated peritoneal mesothelial cells. **a** BRG1 was detected by immunohistochemical staining. Representative micrographs show the expression and localization of BRG1 in parietal peritoneum from PD mice. Scale bar, 50 μm. Saline, saline-injected control group. PDF, PD fluid containing 4.25% glucose-injected group. **b** Western blot analyses of visceral peritoneum expression of BRG1, Fibronectin, a-SMA in different groups are presented. Numbers (1, 2 and 3) represent different samples in a given group. **c** HMrSV5 cells were treated with TGF-β1 (5 ng/ml) for the indicated time period. Western blot analyses of expression of BRG1, Fibronectin, a-SMA in different groups are presented. **d** HMrSV5 cells were exposed to the mixture of 4.25%PD fluid(PDF) and culture medium by 1:4,1:3 and 1:2,respectively. Cells incubated in medium diluted 1:2 with sterile saline served as a control. Western blot analyses of expression of BRG1, Collagen I, TGF-β1, E-cadherin in different groups are presented. Numbers (1, 2 and 3) represent different samples in a given group. **e** Primarily cultured rat peritoneal mesothelial cells(RPMCs) exhibited cobble-like appearance under light microscope. Scale bar, 100 μm. **f** Top: cellular immunofuorescence staining revealed that E-cadherin (red) was positive in primarily cultured RPMCs. Bottom: cellular immunofuorescence staining revealed that Vimentin(green) was positive but a-SMA (red) was nagetive in primarily cultured RPMCs.Scale bar, 50 μm. **g** RPMCs were treated without or with TGF-β1 (5 ng/ml) for 48 h. Western blot analyses of expression of BRG1, Collagen I, Fibronectin, E-cadherin, Vimentin in different groups are presented. Numbers (1, 2 and 3) represent different samples in a given group. **h** RPMCs were exposed to the mixture of 4.25%PDF and culture medium by 1:2. Cells incubated in medium diluted 1:2 with sterile saline served as a control. Western blot analyses of expression of BRG1, Collagen I, E-cadherin, TGF-β1, a-SMA in different groups are presented. Numbers (1, 2 and 3) represent different samples in a given group

To assess the expression of BRG1 in a mouse model of PD, we initially conducted the immunohistochemical staining and found that compared to the saline-injected mice, the expression of BRG1 was notably augmented, particularly in the nuclei, in parietal peritoneal tissues from PD mice (Fig. 1a). In addition, western bolt analysis revealed that in comparison to saline group, the expression of BRG1 was increased in visceral peritoneum from PD mice, along with an accumulation of ECM proteins such as a-SMA and Fibronectin (Fig. 1b and Additional file 1: Fig. S1a).

We then examined the BRG1 expression in vitro stimulation with TGF-β1 or 4.25%PD fluid respectively. TGF-β1 substantially increased synthesis of ECM-related proteins, such as Fibronectin and a-SMA, in HMrSV5 cells (Fig. 1c and Additional file 1: Fig. S1c, S1d). Of note, BRG1 expression was increased after 48 h by TGF-β1 stimulation (Fig. 1c and Additional file 1: Fig. S1b). Consistently, PD fluid also enhanced the expression of BRG1, Collagen I, TGF-β1, while decreasing E-cadherin level in HMrSV5 cells (Fig. 1d and Additional file 1: Fig. S1e–S1h).

We further isolated primary rat peritoneal mesothelial cells (RPMCs) and incubated them with TGF-β1 or PD fluid respectively. Primary cultured RPMCs of the third passage exhibited a polygonal cobblestone-like appearance (Fig. 1e) and expressed both mesenchymal marker (Vimentin) and epithelial marker (E-cadherin), but not myofibroblast marker (a-SMA) (Fig. 1f). Compared to the control group, TGF-β1 stimulation increased the expression of mesenchymal marker Vimentin and profibrotic factors such as Fibronectin and Collagen I, while the level of epithelial marker E-cadherin decreased, indicating the occurrence of MMT in RPMCs (Fig. 1g and Additional file 1: Fig. S1i). Of note, BRG1 was also elevated when exposed to TGF-β1 stimulation (Fig. 1g and Additional file 1: Fig. S1i). Consistently, compared to the saline group, incubation with 4.25%PD fluid augmented the expression of BRG1, Collagen I, a-SMA, TGF-β1, while reducing the expression of E-cadherin (Fig. 1h and Additional file 1: Fig. S1j). These data indicate that the elevation of BRG1 might be involved with peritoneal fibrosis.

### BRG1 promotes fibrotic responses in peritoneal mesothelial cells.

**Fig. 2:**
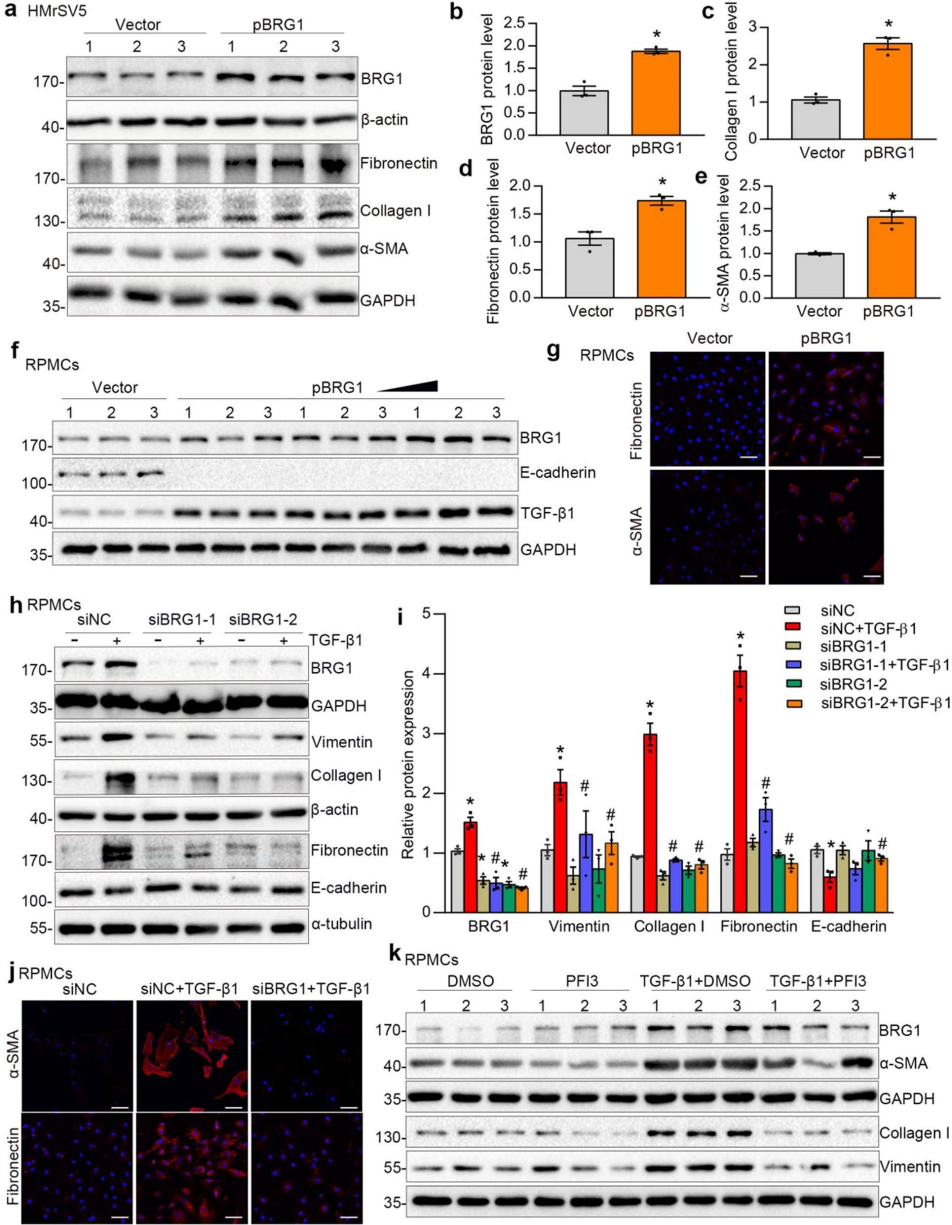
BRG1 promotes fibrotic responses in vitro. **a**–**e** HMrSV5 cells were transfected with control vector (Vector)or BRG1 expression plasmid (pBRG1) for 48 h. Western blot analyses show that overexpression of BRG1 induced the expression of Fibronectin,Collagen I and a-SMA expression. Representative Western blot (**a**) and quantitative data (**b**–**e**) in different groups are presented. Numbers (1, 2 and 3) represent different samples in a given group. *P < 0.05 versus Vector. **f** RPMCs were transfected with control vector (Vector) or BRG1 expression plasmid (pBRG1) for 48 h. Western blot analyses show that overexpression of BRG1 induced the expression of TGF-β1 but reduced the E-cadherin expression. Numbers (1, 2 and 3) represent different samples in a given group. Triangle represents incremental plasmid dosage (2 μg, 3 μg and 4 μg/plate). **g** Representative micrographs show that BRG1 overexpression promoted Fibronectin(top) and a-SMA(bottom) expression. Scale bar, 50 μm. **h**, **i** Western blot analyses show that BRG1 interference by siRNA strategy reduced TGF-β1-induced expression of Collagen I, Vimentin, Fibronectin and restored E-cadherin expression. Representative Western blot (**h**) and quantitative data (**i**) in different groups are presented. *P < 0.05 versus siNC, ^#^P < 0.05 versus siNC in the presence of TGF-β1. **j** Representative micrographs show that knockdown of BRG1 repressed a-SMA (top) and Fibronectin(bottom) expression induced by TGF-β1. Scale bar, 50 μm. **k** Western blot analyses show the effect of BRG1 inhibitor PFI3 on TGF-β1-induced expression of Collagen I, a-SMA, Vimentin. Numbers (1, 2 and 3) represent different samples in a given group

To investigate the role of BRG1 on the fibrotic responses in peritoneal mesothelial cells, HMrSV5 cells were transfected with either a BRG1 expression vector (pBRG1) or an empty vector. RT-qPCR (Additional file 1: Fig. S2a) and western blot analysis (Fig. 2a, b) were employed to evaluate the transfection efficacy. BRG1 overexpression dramatically increased synthesis of ECM-related protein, such as Fibronectin, Collagen I and a-SMA (Fig. 2a–e). We then transfected primary RPMCs with a BRG1 expression vector (pBRG1) or an empty vector, and the transfection efficacy was assessed using RT-qPCR (Additional file 1: Fig. S2b) and western blot analysis (Fig. 2f, Additional file 1: Fig. S2c). Results of western bolt revealed that BRG1 overexpression upsurged the TGF-β1 expression while decreasing E-cadherin expression (Fig. 2f and Additional file 1: Fig. S2c). Immunofluorescence staining additionally confirmed the augmentation of Fibronectin and a-SMA in RPMCs upon BRG1 overexpression (Fig. 2g).

Moreover, we knocked down BRG1 expression using an siRNA strategy and BRG1 inhibitor PFI3. RPMCs were transfected with control (siNC) or BRG1-specific siRNA (siBRG1), followed by exposure to TGF-β1. Efficient BRG1 knockdown was confirmed by RT-qPCR (Additional file 1: Fig. S2d) and western blot analysis (Fig. 2h, i). Exogenous TGF-β1 increased abundance of BRG1 (Fig. 2h, i). By contrast, BRG1 interference markedly abolished the synthesis of Vimentin, Collagen I, Fibronectin induced by TGF-β1 and restored the expression of E-cadherin (Fig. 2h, i). Immunofluorescence staining also confirmed the attenuation of Fibronectin and a-SMA when BRG1 was inhibited in TGF-β1-stimulated cells (Fig. 2j). PFI-3 is a selective chemical probe to target SMARCA bromodomains and impede the action of BRG1 by disrupting its connection with acetylated histone [30]. Results of western blot analysis revealed that PFI-3 largely inhibited the overproduction of Vimentin and Collagen I by TGF-β1 in RPMCs, without influencing the expression of a-SMA (Fig. 2k, Additional file 1: Fig. S2e). These data suggest that BRG1 might be an essential determinant in fibrotic responses of peritoneal mesothelial cells.

### BRG1 activates TGF-β1-Smad2/3 pathway in peritoneal mesothelial cells

**Fig. 3:**
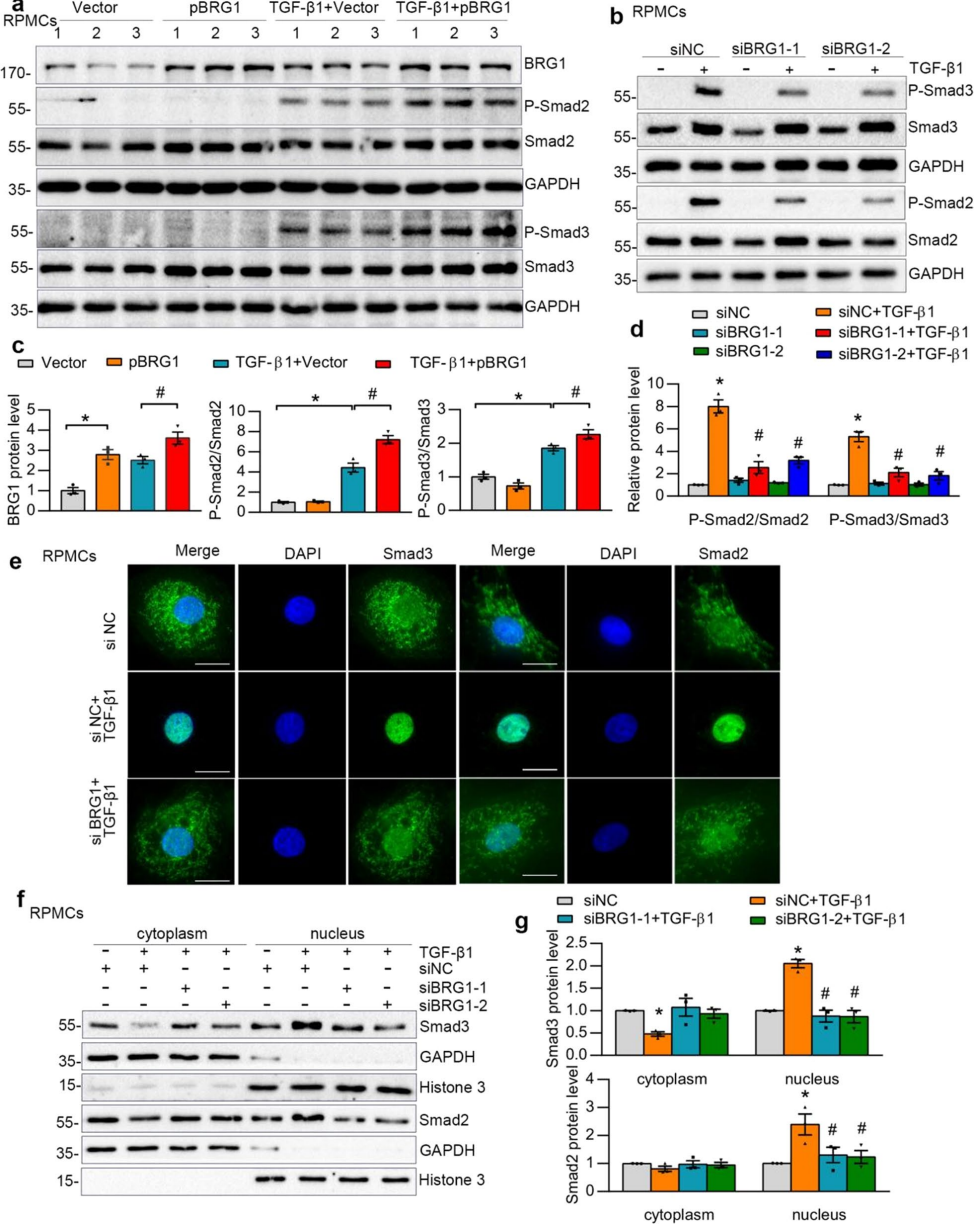
BRG1 activates TGF-β1-Smad2/3 signaling pathway in vitro. **a**, **c** Western blot analyses show that BRG1 overexpression induced phosphorylation of Smad2 and Smad3.Western blot (**a**) and quantitative data (**c**) in different groups are presented. Numbers (1, 2 and 3) represent different samples in a given group. *P < 0.05 versus Vector, ^#^P < 0.05 versus Vector in the presence of TGF-β1. (**b**, **d**) Western blot analyses show that knockdown of BRG1 by siRNA strategy reduced TGF-β1-induced phosphorylation of Smad2 and Smad3. Western blot (**b**) and quantitative data (**d**) in different groups are presented. *P < 0.05 versus siNC, ^#^P < 0.05 versus siNC in the presence of TGF-β1. **e** Representative micrographs show that knockdown of BRG1 repressed Smad2 and Smad3 nuclear accumulation induced by TGF-β1. Scale bar, 10 μm. **f**, **g** Western blot shows the distribution of Smad2 and Smad3 in cytosolic and nuclear fractions of different groups as indicated. GAPDH and Histone H3 were used to normalize cytosolic and nuclear fractions, respectively. Western blot (**f**) and quantitative data (**g**) in different groups are presented. *P < 0.05 versus siNC, ^#^P < 0.05 versus siNC in the presence of TGF-β1

Activation of TGF-β1 signaling is implicated in the development and progression of peritoneal fibrosis [5, 7, 31]. Data from Fig. 3a, c revealed that overexpression of BRG1 amplified TGF-β1-increased-phosphorylation of Smad2 and Smad3. Moreover, Smad2 and Smad3 became phosphorylated and activated with TGF-β1 in RPMCs, yet this process is impeded by knockdown on BRG1, as illustrated in Fig. 3b, d. Immunofluorescent staining revealed that following TGF-β1 stimulation, Smad2 and Smad3 were accumulated in the nuclei of RPMCs (Fig. 3e). However, BRG1 inhibition largely abolished the TGF-β1-triggered Smad2 and Smad3 nuclear accumulation (Fig. 3e). We also explored the distribution of Smad2 and Smad3 between cytoplasm and nucleus. Western blot results revealed the nuclear localisation of Smad2 and Smad3 in RPMCs exposed to TGF-β1 was impeded when BRG1 was inhibited (Fig. 3f, g). However, although TGF-β1 induced the phosphorylation of ERK1/2, P38 MAPK and JNK, knockdown of BRG1 had no impact on these molecules in RPMCs (Additional file 1: Fig. S3).

### An anti-fibrotic compound MCL diminishes BRG1-induced fibrotic responses and inhibits TGF-β1-Smad2/3 signaling pathway in vitro

**Fig. 4:**
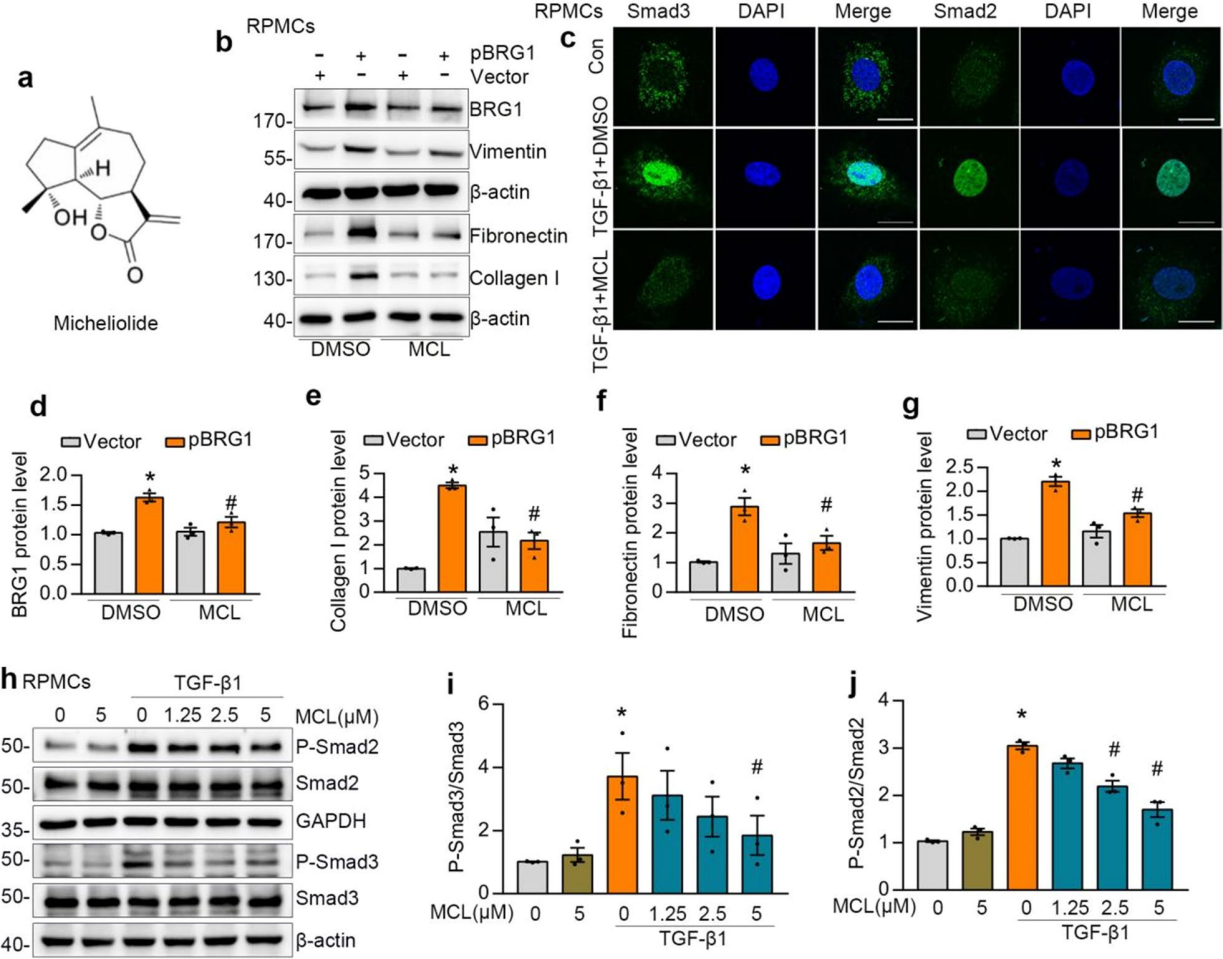
MCL inhibits BRG1-induced fibrotic responses and TGF-β1-Smad2/3 pathway in vitro. **a** The structure of MCL. **b**, **d**, **e**, **f**, **g** Western blot analyses show that MCL reduced BRG1-induced expression of Vimentin, BRG1, Collagen I and Fibronectin in RPMCs. Representative Western blot (**b**) and quantitative data on the relative abundance of proteins (**d**–**g**) in different groups are presented. *P < 0.05 versus Vector. ^#^P < 0.05 versus pBRG1. **c** Representative micrographs show that MCL reduced Smad3 and Smad2 nuclear accumulation induced by TGF-β1. Scale bar, 10 μm. **h**–**j** Western blot analyses show that MCL reduced TGF-β1-induced phosphorylation of Smad2 and Smad3. Representative Western blot (**h**) and quantitative data (**i**, **j**) in different groups are presented. *P < 0.05 versus conrol group, ^#^P < 0.05 versus TGF-β1 group

Given that the potential pro-fibrotic role of BRG1 in TGF-β1-stressed peritoneal mesothelial cells, we further explore the possibility of targeting BRG1 in the intervention of peritoneal fibrosis. Our previous study has uncovered that MCL is an effective anti-fibrotic compound in a mouse model of peritoneal fibrosis and in TGF-β1-stimulated HMrSV5 cells [27]. In this study, we explored the effect of MCL in BRG1- induced fibrotic responses. Interestingly, western blot results revealed that BRG1 overexpression significantly promoted the Vimentin, Fibronectin, Collagen I synthesis, which was remarkably reversed upon MCL treatment (Fig. 4b–g). Besides, TGF-β1 induced the phosphorylation of Smad2 and Smad3, whereas MCL hampered this process (Fig. 4h–j). Immunofluorescent staining revealed that Smad2 and Smad3 were accumulated in the nuclei after TGF-β1 stimulation, which were abolished by MCL treatment (Fig. 4c). Moreover, BRG1 overexpression increased the expression of TGF-β1 and phosphorylation of Smad3. Nevertheless, MCL abolished the BRG1-triggered TGF-β1 expression and Smad3 phosphorylation (Fig. 7i, Additional file 1: Fig. S4e, S4f). These data above imply MCL might be an anti-fibrotic compound by targeting BRG1.

### Administration of DMAMCL inhibited BRG1 overexpression-induced peritoneal fibrosis and TGF-β1-Smad2/3 pathway in a mouse PD model

**Fig. 5:**
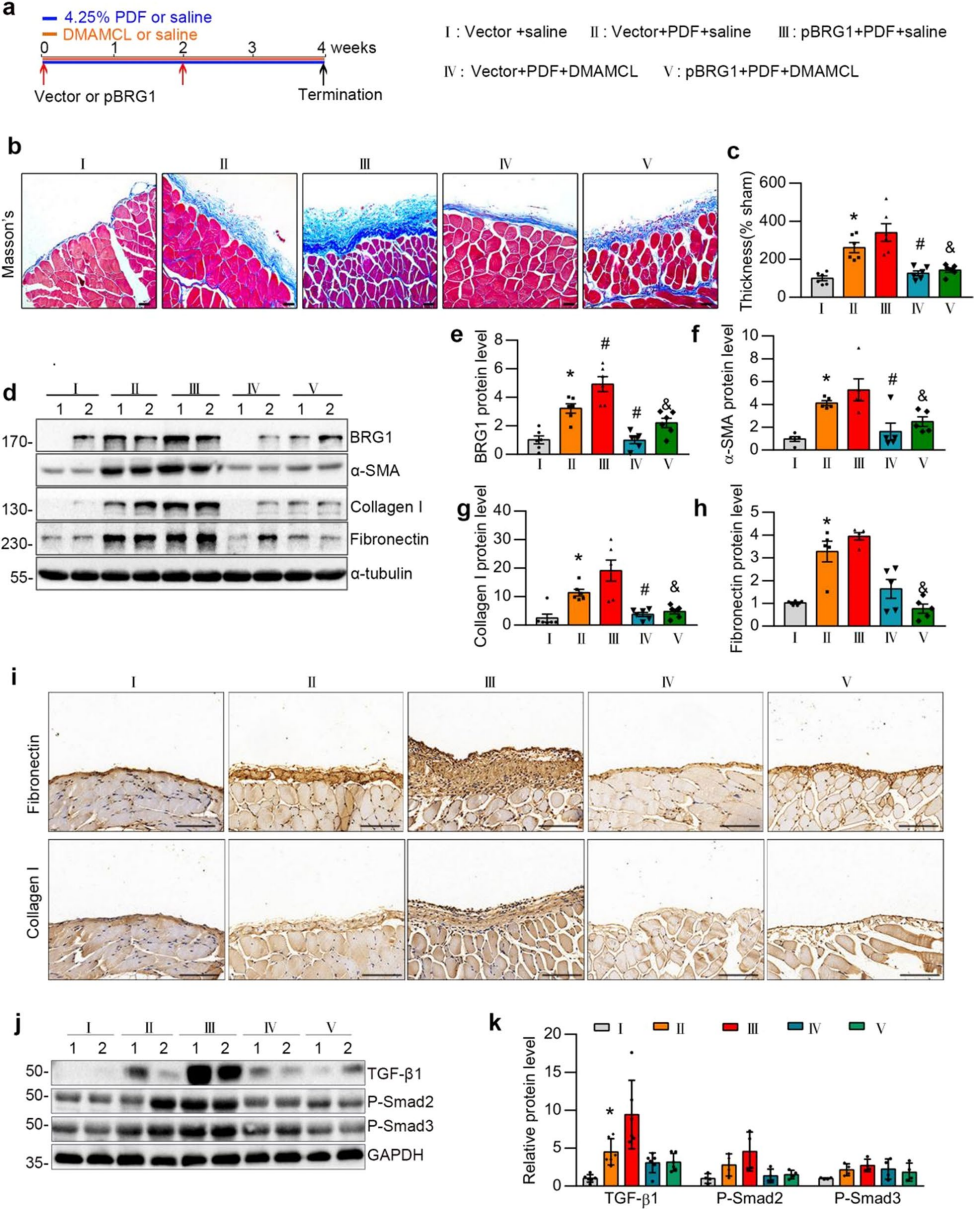
Administration of DMAMCL ameliorates BRG1 overexpression-induced peritoneal fibrosis in a mouse model of PD. **a** Experimental design. Red arrows indicate the injection of control (Vector) or BRG1 overexpression plasmid (pBRG1). Orange line indicates the administration of DMAMCL or saline. **b**, **c** Representative micrographs show collagen deposition by Masson’s trichrome staining (**b**) and quantitative analysis (**c**) in the parietal peritoneum in different groups as indicated. Scale bar, 50 μm. *P < 0.05 versus group I (vector + saline), ^#^P < 0.05 versus group II (vector + PDF + saline). ^&^P < 0.05 versus group III (pBRG1 + PDF + saline). n = 5–6 per group. **d**–**h** Western blot analysis showed visceral peritoneum expression of BRG1, Fibronectin, Collagen I, and a-SMA in different groups as indicated. Representative Western blot (**d**) and quantitative data on the relative abundance of BRG1 (**e**), a-SMA (**f**), Collagen I (**g**), and Fibronectin (**h**) proteins are presented. *P < 0.05 versus group I (vector + saline), ^#^P < 0.05 versus group II (vector + PDF + saline). ^&^P < 0.05 versus group III (pBRG1 + PDF + saline). n = 5–6 per group. **i** Representative micrographs show Fibronectin (top), and Collagen I (bottom) staining in different groups as indicated. Fibronectin and Collagen I were detected by immunohistochemical staining. Scale bar, 50 μm. **j**–**k** Western blot analysis showed visceral peritoneum expression of TGF-β1,P-Smad2 and P-Smad3 in different groups as indicated. Representative Western blot (**j**) and quantitative data (**k**) on the relative abundance of TGF-β1, P-Smad2 and P-Smad3 are presented. *P < 0.05 versus group I(vector + saline), ^#^P < 0.05 versus group II(vector + PDF + saline). ^&^P < 0.05 versus group III (pBRG1 + PDF + saline). n = 4–6 per group

In vivo, to further investigate the functional role of DMAMCL, the pro drug of MCL, in BRG1-induced peritoneal fibrosis, mice undergoing daily intraperitoneal injection of PD fluid were subjected to BRG1 overexpression plasmid through ultrasonic microbubbles at day 0 and day 14. Subsequently, these mice were treated with daily DMAMCL or saline. The efficiency of ultrasound-microbubble-mediated BRG1 overexpression in mice was shown by western blot analysis (Fig. 5d). Masson’s trichrome staining showed that compared to the normal group, the PD mice had thicker matrix deposition and mice that underwent PD and overexpression of BRG1 had thickest peritoneal fibrous layer among the five groups (Fig. 5b, c). DMAMCL alleviated the PD-induced matrix deposition and remarkably rescued the matrix deposition upon BRG1 overexpression (Fig. 5b, c). Synthesis of ECM-related proteins like a-SMA, Collagen I, Fibronectin were higher in PD mice and the most abundant in BRG1-overexpression-PD mice. DMAMCL repressed these ECM-related proteins level and upon BRG1 overexpression, reversed their expression (Fig. 5d–h). The similar results of Fibronectin and Collagen I synthesis were observed by immunohistochemical staining (Fig. 5i). Moreover, PD fluid exposure increases TGF-β1 expression and its downstream signaling, which was manifested by increasing the phosphorylation of Smad2 and Smad3 in PD mice. This effect was further aggravated upon BRG1 overexpression. Nevertheless, DMAMCL administration repressed TGF-β1-Smad2/3 signaling and reversed TGF-β1-Smad2/3 pathway when BRG1 was overexpressed.

### BRG1 interacts with H3K14ac to promote fibrotic responses, whereas MCL treatment disrupts their interactions

**Fig. 6:**
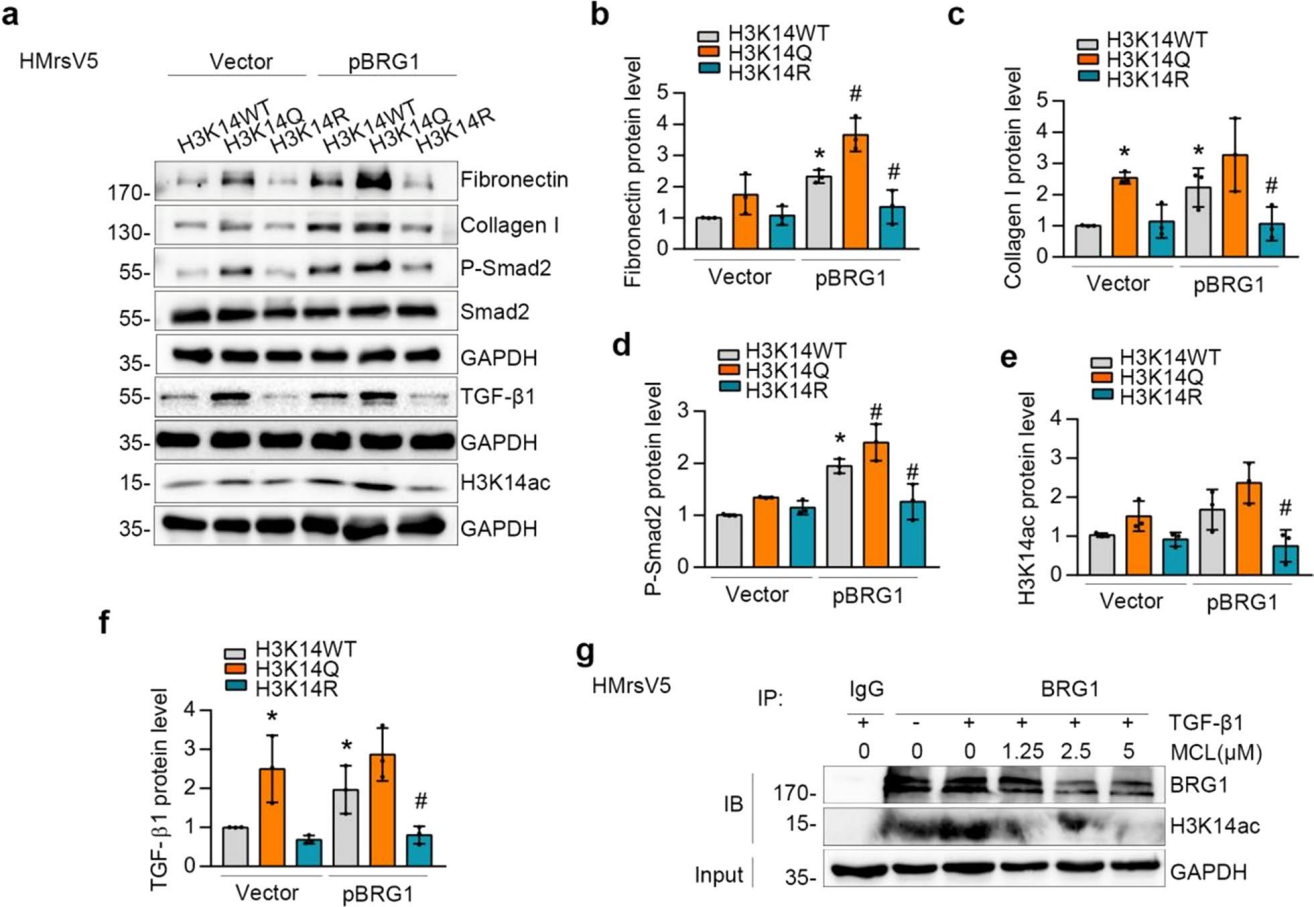
BRG1-H3K14ac complex plays a crucial role in fibrotic responses, and MCL disrupts this complex. **a**–**f** BRG1 expression vector and H3K14 different mutation vectors were co-transfected into HMrSV5 cells. Lysine 14 was mutated into glutamine to mimic acetylation and mutated into arginine to inhibit acetylation, named as H3K14Q or H3K14R, respectively. Western blot analyses (**a**) and quantitative data of Fibronectin (**b**), Collagen I (**c**), P-Smad2 (**d**), H3K14ac (**e**) and TGF-β1 (**f**) in different groups are presented. *P < 0.05 versus vector, #P < 0.05 versus pBRG1. **g** Co-immunoprecipitation demonstrated that BRG1 interacted with H3K14ac in HMrSV5 cells, which was blocked by MCL. Cells were incubated with TGF-β1 with or with DMSO or different concentration of MCL for 48 h. Cell lysates were IP with anti-BRG1, followed by IB with anti-BRG1 and anti-H3K14ac

We further delve deeper into the mechanism of MCL in related to BRG1-induced fibrotic responses. As previous studies showed, the binding of BRG1 to acetylated histones H3K14ac is a crucial factor in its gene regulation [32, 33]. The role of BRG1-H3K14ac complex in fibrotic responses was explored in vitro. HMrSV5 cells were co-transfected with BRG1 expression vector and H3K14 different mutation vectors, in which lysine 14 was mutated into glutamine (H3K14Q) to mimic acetylation and mutated into arginine (H3K14R) to inhibit acetylation. H3K14Q mutant augmented the expression of Fibronectin and Collagen I, which was not observed in H3K14R mutant. Moreover, BRG1 overexpression further promoted the expression of Fibronectin and Collagen I when co-transfected with H3K14Q, whereas H3K14R co-transfection rescued this effect (Fig. 6a–e). Furthermore, the increase of TGF-β1 by BRG1 was further augmented in H3K14Q mutant, while it was abolished in H3K14R mutant (Fig. 6a, f). Additionally, BRG1-induced Smad2 phosphorylation was further elevated in H3K14Q mutant, whereas it was blocked in H3K14R mutant (Fig. 6a, d). Subsequently, we examined the influence of MCL on the binding of BRG1 and H3K14ac. Interestingly, co-immunoprecipitation analysis showed the BRG1-H3K14ac interaction was abolished by incubation with MCL (Fig. 6g), suggesting that MCL’s ability to prevent peritoneal fibrosis may be through disrupting the BRG1 interaction with H3K14ac.

### MCL assuages BRG1-induced fibrotic responses by directly binding to the residue of asparagine 1540 of BRG1

**Fig. 7:**
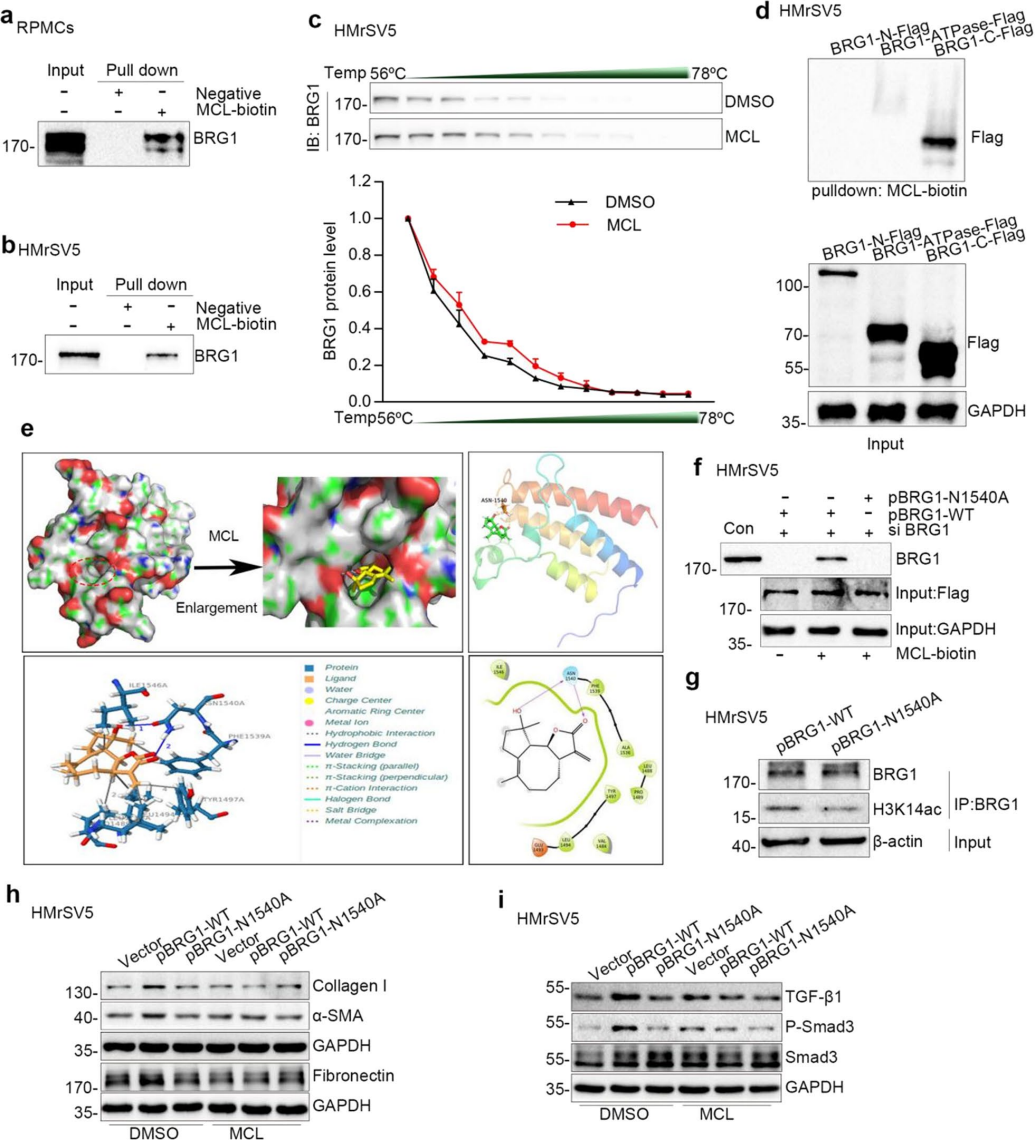
MCL assuages BRG1-induced fibrotic responses by binding to the residue of asparagine 1540 of BRG1. **a** Pull-down experiment with a biotin-MCL indicated that MCL directly bound to BRG1 in RPMCs lysates. **b** Pull-down experiment with a biotin-MCL indicated that MCL directly bound to BRG1 in HMrSV5 cell lysatases. **c** Cellular thermal shift assay confirmed the binding of MCL to BRG1 in HMrSV5 cells. **d** Pull-down experiment with a biotin-MCL indicated that MCL directly bound to BRG1 C-terminal region. **e** Molecular docking of MCL with the protein BRG1. **f** Pull-down experiment with a biotin-MCL indicated that BRG1 N1540A mutant abrogated the interaction between MCL and BRG1. **g** Co-immunoprecipitation demonstrated that the interaction between BRG1 and H3K14ac was decreased after the mutation of BRG1 N1540. After transfection with BRG1 full length plasmid or N1540A mutant plasmid, cell lysates were IP with anti-BRG1, followed by IB with anti-BRG1 and anti-H3K14ac. **h**, **i** Cells were transfected with BRG1 full length plasmid, N1540A mutant plasmid or vector plasmid, and then were treated with DMSO or MCL. Western blot analyses of expression of a-SMA, Collagen I, Fibronectin, TGF-β1 and P-Smad3 in different groups are presented

To explore the mechanism of MCL disrupting BRG1 interaction with H3K14ac, cell lysates were incubated with MCL-biotin probe or negative probe, and mixtures were separately pulled down with streptavidin-coated agarose beads, followed by western blot analysis. Pull down analysis validated the interaction between MCL and BRG1 in both HMrSV5 cells lysates and RPMCs lysates (Fig. 7a, b). We next performed cellular thermal shift assay to confirm MCL binding to BRG1 (Fig. 7c). BRG1 is composed of multiple domains, most of which have been identified by primary sequence analysis and include an conserved catalytic ATPase domain, as well as a conserved C-terminal bromodomain, AT-hook motif and the N-terminal region housing QLQ, HSA and BRK domains[34]. To next investigate the direct bind site between MCL and BRG1, HMrSV5 cells were transfected with three BRG1 domain deletion mutants (N-terminal domain (1–765), C-terminal domain (1301–1647) or ATPase domain (766–1300)) (Additional file 1: Fig. S4a) and cells lysates were incubated with MCL-biotin probe. Pull-down assay showed that MCL interacted with C-terminal domain of BRG1 (Fig. 7d). And the predicted conformation of MCL-bound human BRG1 protein was further evaluated by computational modeling analysis (Fig. 7e). Of note, MCL has the potential to interact with the conserved asparagine 1540 residue (N1540) in the C-terminal bromodomain of BRG1 via two traditional hydrogen bonds, by mutating the N1540 of BRG1 to alanine (N1540A), we ascertained that asparagine 1540 of BRG1 was indispensable for its interaction with MCL. This was confirmed by the data that the BRG1 N1540A mutant completely eliminated the interaction between MCL and BRG1 (Fig. 7f). Furthermore, co-immunoprecipitation analysis showed that the interaction between BRG1 and H3K14ac was decreased after the mutation of BRG1 N1540 (Fig. 7g). These suggest that MCL disrupt interaction with H3K14ac may through its interaction with N1540 of BRG1.

Western bolt analysis revealed that, unlike BRG1 overexpression group, which promoted synthesis of Fibronectin, Collagen I, a-SMA, and activated TGF-β1-Smad3 signaling, the BRG1 N1540A mutant was unable to produce these effects (Fig. 7h-7i, Additional file 1: Fig. S4b–g). Moreover, while MCL treatment assuaged the effects of BRG1-induced-ECM deposition and TGF-β1-Smad3 signaling, the mutation of BRG1 N1540A prevented any further effects of MCL (Fig. 7h, i, Additional file 1: Fig. S4b–g). Altogether, these data above indicated that MCL inhibited BRG1-induced ECM deposition and TGF-β1-Smads signaling partially by binding directly to BRG1 at the N1540 residue.

## Discussion

**Fig. 8:**
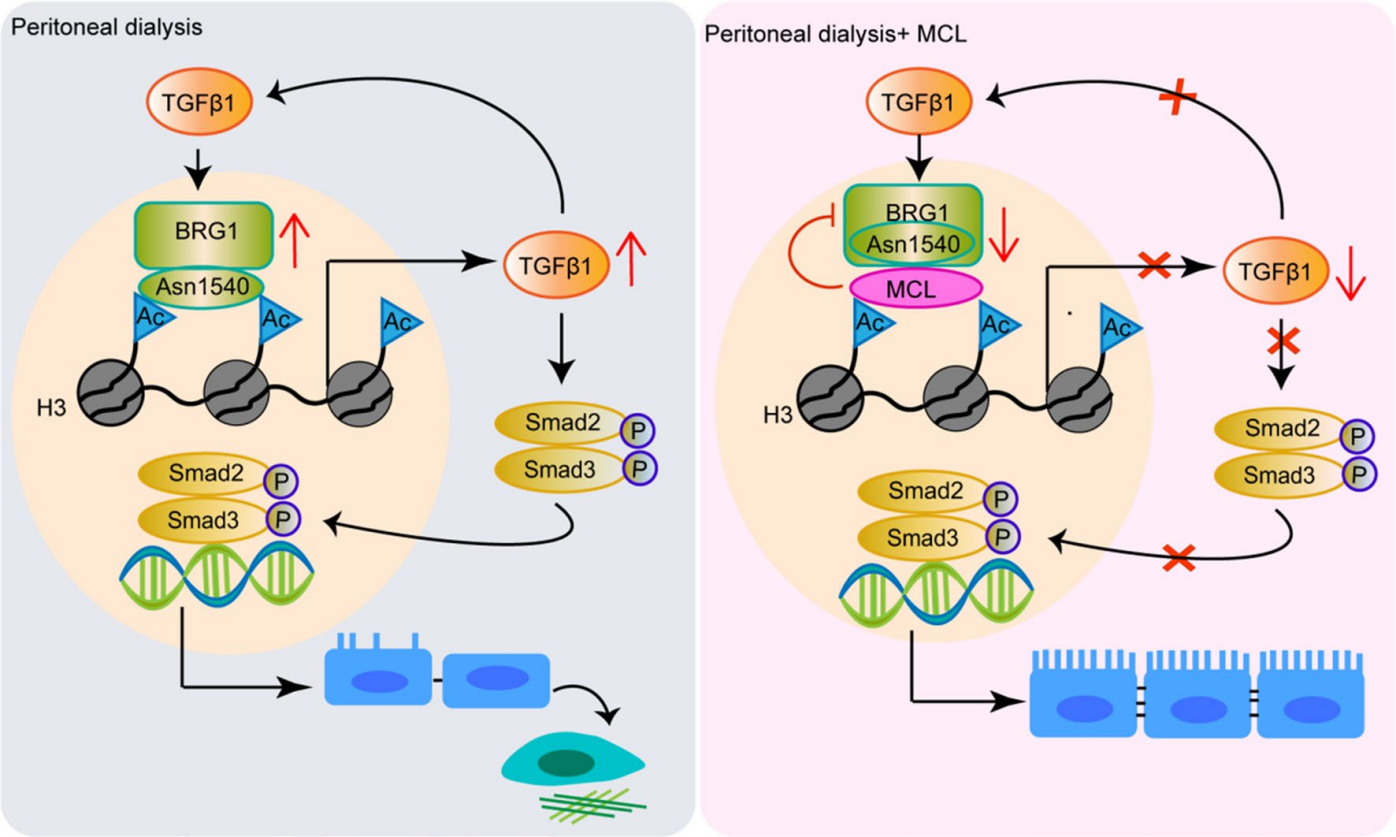
Schematic diagram of MCL’s protection against PD-related peritoneal fibrosis. MCL binds to the conserved N1540 residue of BRG1 and disrupts interaction of BRG1-H3K14ac complex, thereby leading to inhibit TGF-β1-Smad2/3 signaling pathway and fibrotic responses in peritoneal mesothelial cells

In this study, we first identify chromatin remodeling protein BRG1 as an important pro-fibrotic mediator in peritoneal fibrosis. The expression of BRG1 is aberrantly elevated in a mouse PD model and in peritoneal mesothelial cells exposed to TGF-β1 or PD fluid, and is linked to TGF-β1-Smad2/3 signaling pathway and peritoneal fibrosis. More importantly, we reveal that MCL specifically binds to the conserved asparagine 1540(N1540) residue of BRG1, thereby disrupting the recognition and binding of BRG1 to H3K14ac. This ultimately inhibits BRG1-induced fibrotic responses and TGF-β1-Smad2/3 signaling pathway. These data suggest that MCL may be a natural lead compound of BRG1-targeted therapeutic strategy to prevent and treat peritoneal fibrosis (Fig. 8).

Peritoneal fibrosis is a common complication of PD, which in the long run, results in ultrafiltration failure and the discontinuation of PD therapy [6, 7]. Both high-glucose PD solution and TGF-β1 are fibrogenic factors during the process of peritoneal fibrosis [8, 10, 31, 35, 36]. In this study, we reveal that BRG1 is dramatically elevated in a mouse model of PD and in PD fluid-treated or TGF-β1-strssed cells. Either treatment with siRNA-mediated BRG1 knockdown or BRG1 inhibitor PFI3 blocks TGF-β1-induced fibrotic responses (Fig. 2), suggesting that BRG1 may act as a positive mediator of fibrotic responses downstream of TGF-β1. Consistent with our study, Gong et al. [16], Niu et al. [37], Yang et al. [38] have found that BRG1 is a downstream of TGF-β1 in various cell types. Of note, we also notice that the mRNA level of BRG1 cannot be augmented by TGF-β1 stimulation (data not shown), implying that TGF-β1 might regulate the expression of BRG1 in a post-transcriptional pathway manner in peritoneal mesothelial cells. These deserve comprehensive investigation in the future. Besides, in this study we present an intriguing reciprocal activation loop, wherein TGF-β1 expression could be augmented by BRG1 overexpression (Fig. 2、Fig. 7j). This TGF-β1-BRG1 positive feedback loop can also be detected in renal tubular epithelial cells [16]. It may be plausible that BRG1 plays profibrotic roles in peritoneal mesothelial cells by the amplification of the TGF-β1 signaling pathway.

TGF-β1 signaling is considered as a crucial canonical molecular pathway of peritoneal fibrosis, including TGF-β1-Smads and TGF-β1-non-Smads pathway [5, 7, 31]. Previous studies have implicated that TGF-β1-Smads signaling pathway could be activated by BRG1 in some biological systems. Li et al. indicate that BRG1 promotes liver fibrosis through TGF-β-Smads signal pathway [39]. Data shown by Ross et al. reveal that BRG1 can interact with Smad2 from TGF-β-induced nuclear extracts [40]. In this study, we observe that knockdown of BRG1 inhibits phosphorylation and activation of Smad2 and Smad3, while overexpression BRG1 phosphorylates and activates both of Smad2 and Smad3 (Fig. 3), suggesting that BRG1 promotes peritoneal fibrosis, can be, at least in part, attributed to activation of the TGF-β1-Smad2/3 pathway. Of note, BRG1 fail to phosphorylate and activate P38 MAPK, ERK,JNK pathways, three common well-known TGF-β1-non-Smads signaling [11], suggest that BRG1 promotes peritoneal fibrosis through Smads dependent signaling pathway. Even so, we cannot neglect the effects of TGF-β1-Smads-independent pathways during peritoneal fibrosis [7, 41, 42].

Another valuable finding in this study is that MCL, a novel guaianolide sesquiterpene lactone semisynthesized from parthenolide [23, 24], prevents and ameliorates peritoneal fibrosis, as demonstrated in our study previously [27], through impeding BRG1 from recognizing and attaching to H3K14ac by binding to the N1540 of BRG1. BRG1 is the central catalytic ATPase of the switch/sucrose nonfermentable chromatin remodeling complex, containing a bromodomain that has been shown to anchor the entire complex to promoter nucleosomes by interacting with histones that are acetylated at specific lysine residues [43, 44]. Among that, H3K14ac is a common target for BRG1 recruitment in order to modulate gene regulation [32, 33]. In our studies, co-immunoprecipitation analysis reveals that BRG1-H3K14ac complex exists in peritoneal mesothelial cells. However, when MCL binds to BRG1, it disrupts the interaction between BRG1 and H3K14ac, thereby suppressing the fibrotic responses and TGF-β1-Smad2/3 signaling pathway that is triggered by BRG1-H3K14ac complex (Fig. 6). Our results provide the evidence that histone acetylation can function as a signal for recruiting specific proteins to modulate TGF-β1-Smad2/3 signaling pathway, yet the target genes directly regulated by the BRG1-H3K14ac complex has not been clarified. These deserve comprehensive investigation in the future.

We further decipher the relationship between MCL and BRG1. Through MCL-biotin pulldown analysis, cellular thermal shift assay, BRG1 site mutation technology, and molecular docking experiments, we confirm that MCL binds to N1540 residue of BRG1. After the mutation of N1540 to alanine (N1540A), MCL fails to bind to BRG1 and thus, unsuccessful in suppressing BRG1-induced fibrotic responses and TGF-β1-Smad2/3 signaling pathway (Fig. 7). These data suggest that N1540 residue of BRG1 is a significant site for the interaction between MCL and BRG1 in the context of peritoneal fibrosis. Consistent with our research, Shen et al. shows a hydrogen bond between the carbonyl group of N-acetyl in H3K14ac and the side chain amide in N1540 of BRG1 by a molecular dynamics simulation model, revealing the potential interactions BRG1 bromodomain for histone H3K14ac [43]. In addition, Paul et al. reveal that bromodomain of caenorhabditis elegans BRG1 can recognize H3K14ac [33]. Furthermore, BRG1 can act as a scaffold to recruit various coactivators and corepressors, or histone deacetylase and acetyltransferaseis, to bind and recognize histones acetylation for modulating target genes [45, 46]. For example, BRG1 is recruited to Brd4-P300 interaction to mediates H3K27 and H3K56 acetylation at pluripotent genes [45]. BRG1 binds to gamma-H2AX nucleosomes by interacting with acetylated H3 o facilitate DNA double-strand break repair [47]. Therefore, transcriptional cofactors that BRG1 bind to recognize H3K14ac need further research.

Currently there are few safe and effective therapeutic approaches for the treatment of peritoneal fibrosis. MCL may be a potential agent for the prevention and alleviation of peritoneal fibrosis because: (1) the drug/prodrug form of MCL/DMAMCL had at least three advantages over PTL: greater stability of active drug, reduced toxicity to normal cells, and more sustained release of active drug from the prodrug from. [25, 29, 48]; (2) it has been reported to possess anti-inflammatory, anti-fibrotic, antioxidant, and anti-aging properties [26, 27, 48–51]. Of note, it prevents and ameliorates peritoneal fibrosis in a mouse model of peritoneal fibrosis and in TGFβ1-treated peritoneal mesothelial cells as illustrated by our previous study [27]; and (3) we present compelling evidence that BRG1 is a pro-fibrotic regulator during peritoneal fibrosis, yet MCL binds to BRG1, thereby inhibiting BRG1-induced peritoneal fibrosis. However, despite these findings, challenges still exist. MCL can simultaneously regulate multiple targets [29, 48, 52] and might be involved in complicated feedback mechanisms, therefore, more experimental PD animal models, including chlorhexidine gluconate-induced peritoneal fibrosis model [53, 54] and rat model with chronic renal failure resulting from 5/6 nephrectomy [55] are needed to corroborate its efficacy in peritoneal fibrosis. Moreover, safety and effectiveness clinical trials should be conducted to assess the use of MCL in this context. Besides, in recent years, the potential of green nanomaterials to deliver pharmaceuticals to tissue has been widely explored [56, 57], suggesting that further investigation into MCL using nanomaterials to target peritoneal tissue is necessary.

## Conclusions

In summary, we herein demonstrate that aberrant expression of BRG1 in peritoneal mesothelial cells promotes peritoneal fibrosis and activates TGF-β1-Smad2/3 signaling pathway. MCL impeded BRG1 from recognizing and attaching to H3K14ac by binding to the asparagine (N1540) of BRG1, in thus restraining fibrotic responses and TGF-β1-Smad2/3 signaling pathway. Our discovery provides a potential pharmacological mechanism for PD-associated peritoneal fibrosis. It is hopeful that MCL can be developed into a viable therapy for those suffering from PD related-peritoneal fibrosis.

### Supplementary Information

**Additional file 1: Figure S1.** BRG1 is increased in a mouse model of PD and in peritoneal mesothelial cells with TGF-β1 or PD fluid stimulation. **a** Quantitative data of Western blot analyses show the visceral peritoneum expression of BRG1, Fibronectin, a-SMA. *P<0.05 versus saline mice.n=6 per group. **b**–**d** Quantitative data of Western blot analyses show the expression of BRG1 (**b**), Fibronectin (**c**), a-SMA (**d**) in HMrSV5 cells challenged by TGF-β1 for 0, 12, 24 and 48 hours, respectively. *P<0.05 versus time zero. **e**–**h** Quantitative data of Western blot analyses show the expression of BRG1 (**e**), Collagen I (**f**), E-cadherin (**g**) and TGF-β1 (**h**) in HMrSV5 cells stimulated by PDF. *P<0.05 versus saline. **i** Quantitative data of Western blot analyses show the expression of BRG1, Collagen I, E-cadherin, Vimentin,and Fibronectin in RPMCs stimulated by TGF-β1. *P<0.05 versus control group. **j** Quantitative data of Western blot analyses show the expression of BRG1, Collagen I, E-cadherin, TGF-β1, and a-SMA in RPMCs stimulated by PDF. *P<0.05 versus saline. **Figure S2.** BRG1 promotes fibrotic responses in vitro. **a** Graphic presentation shows the mRNA expression of BRG1 in HMrSV5 cells in different groups as indicated. The mRNA level of BRG1 was detected by Quantitative real-time PCR. *P<0.05 versus Vector. **b** Graphic presentation shows the mRNA expression of BRG1 in RPMCs in different groups as indicated.*P<0.05 versus Vector. **c** Quantitative data of Western blot analyses show the expression of BRG1, E-cadherin,TGF-β1. Triangle represents incremental plasmid dosage (2ug,3ug and 4ug/plate). *P<0.05 versus Vector. **d** Graphic presentation shows the mRNA expression of BRG1 in different groups as indicated in RPMCs.*P<0.05 versus Vector. **e** Quantitative data of Western blot analyses show the expression of BRG1, Vimentin, a-SMA, Collagen I in different groups are presented. *P<0.05 versus siNC, ^#^P<0.05 versus siNC in the presence of TGF-β1. **Figure S3.** BRG1 inhibition has no effect on phosphorylation of ERK1/2, P38 MAPK and JNK in vitro. **a**, **b** Western blot analyses show that BRG1 inhibition by siRNA had no effect on phosphorylation of ERK1/2. Representative Western blot (**a**) and quantitative data (**b**) in different groups are presented. **c**, **d** Western blot analyses show that BRG1 inhibition had no effect on P38 phosphorylation. Representative Western blot (**c**) and quantitative data (**d**) in different groups are presented.** e**, **f** Western blot analyses show that BRG1 inhibition had no effect on phosphorylation of JNK. Representative Western blot (**e**) and quantitative data (**f**) in different groups are presented. **Figure S4.** The role of MCL on BRG1-induced fibrotic responses. **a** Schematic representation of BRG1 mutants. BRG1 was divided into three regions: a ATPase domain,a N-terminal region, and a C-terminal region. Each mutant expression plasmids contains a C-terminal flag epitope tag. **(b)**Western blot analyses show that expression of Flag after BRG1 full length or BRG1 N1540A mutant transfection. **c–g** Quantitative data of Western blot analyses show the expression of Collagen I (**c**), Fibronectin (**d**), P-Smad3 (**e**), TGF-β1 (**f**) and a-SMA (**g**) in different groups are presented. *P<0.05 versus Vector.#P<0.05 versus pBRG1. **Table S1.** The detail information of antibodies used in this study. **Table S2.** The sequence of siRNA. **Table S3.** RT-qPCR primer sequences.

## Data Availability

Due to the privacy policy, the datasets analyzed in this study are not publicly available, but they are available from the corresponding author upon reasonable request.

## Abbreviations

PD: Peritoneal dialysis
MCL: Micheliolide
BRG1: Brahma-related gene 1
MMT: Mesothelial-to-mesenchymal transition
α-SMA: α-Smooth muscle actin
TGF-β1: Transforming growth factor-β1
PTL: Parthenolide
DMAMCL: Dimethylaminomicheliolide
RPMCs: Rat peritoneal mesothelial cells
DAPI: 4’,6-Diamidino-2-phenylindole
ECM: Extracellular matrix
H3K14ac: Histone H3 lysine 14 acetylation
