# Supplementary material for: Brahma-related gene 1 acts as a profibrotic mediator and targeting it by micheliolide ameliorates peritoneal fibrosis
Source: J Transl Med. 2023 Sep 19;21:639. doi: 10.1186/s12967-023-04469-w (PMC10510267; doi:10.1186/s12967-023-04469-w)
Supplement: Supplementary file 1 — Additional file 1: Figure S1. BRG1 is increased in a mouse model of PD and in peritoneal mesothelial cells with TGF-β1 or PD fluid stimulation. a Quantitative data of Western blot analyses show the visceral peritoneum expression of BRG1, Fibronectin, a-SMA. *P<0.05 versus saline mice.n=6 per group. b–d Quantitative data of Western blot analyses show the expression of BRG1 (b), Fibronectin (c), a-SMA (d) in HMrSV5 cells challenged by TGF-β1 for 0, 12, 24 and 48 hours, respectively. *P<0.05 versus time zero. e–h Quantitative data of Western blot analyses show the expression of BRG1 (e), Collagen I (f), E-cadherin (g) and TGF-β1 (h) in HMrSV5 cells stimulated by PDF. *P<0.05 versus saline. i Quantitative data of Western blot analyses show the expression of BRG1, Collagen I, E-cadherin, Vimentin,and Fibronectin in RPMCs stimulated by TGF-β1. *P<0.05 versus control group. j Quantitative data of Western blot analyses show the expression of BRG1, Collagen I, E-cadherin, TGF-β1, and a-SMA in RPMCs stimulated by PDF. *P<0.05 versus saline. Figure S2. BRG1 promotes fibrotic responses in vitro. a Graphic presentation shows the mRNA expression of BRG1 in HMrSV5 cells in different groups as indicated. The mRNA level of BRG1 was detected by Quantitative real-time PCR. *P<0.05 versus Vector. b Graphic presentation shows the mRNA expression of BRG1 in RPMCs in different groups as indicated.*P<0.05 versus Vector. c Quantitative data of Western blot analyses show the expression of BRG1, E-cadherin,TGF-β1. Triangle represents incremental plasmid dosage (2ug,3ug and 4ug/plate). *P<0.05 versus Vector. d Graphic presentation shows the mRNA expression of BRG1 in different groups as indicated in RPMCs.*P<0.05 versus Vector. e Quantitative data of Western blot analyses show the expression of BRG1, Vimentin, a-SMA, Collagen I in different groups are presented. *P<0.05 versus siNC, #P<0.05 versus siNC in the presence of TGF-β1. Figure S3. BRG1 inhibition has no effect on phosphorylation of ER [file 12967_2023_4469_MOESM1_ESM.docx]

**Brahma-related gene 1 acts as a profibrotic mediator and targeting it by micheliolide ameliorates peritoneal fibrosis**

Shuting Li^1*^, Congwei Luo^1*^, Sijia Chen^2*^, Yiyi Zhuang^1^, Yue Ji^1^, Yiqun Zeng^1^, Yao Zeng^1^, Xiaoyang He^1^, Jing Xiao^1^, Huizhen Wang^1^, Xiaowen Chen^1#^，Haibo Long^1#^, Fenfen Peng^1#^

^1^Department of Nephrology, Zhujiang Hospital, Southern Medical University, Guangzhou, China

^2^Department of Nephrology and Rheumatology, The First Hospital of Changsha, Changsha, China

^*^These authors contributed equally to this work.

^#^Corresponding author:

Fenfen Peng, Department of Nephrology, Zhujiang Hospital, Southern Medical University, Guangzhou, 510280, China, E-mail: doctorpff@163.com

Haibo Long, Department of Nephrology, Zhujiang Hospital, Southern Medical University, Guangzhou, 510280, China, E-mail: [longhb1966@163.com](mailto:longhb1966@163.com)

Xiaowen Chen, Department of Nephrology, Zhujiang Hospital, Southern Medical University, Guangzhou, 510280, China, E-mail: [573281676@qq.com](mailto:573281676@qq.com)


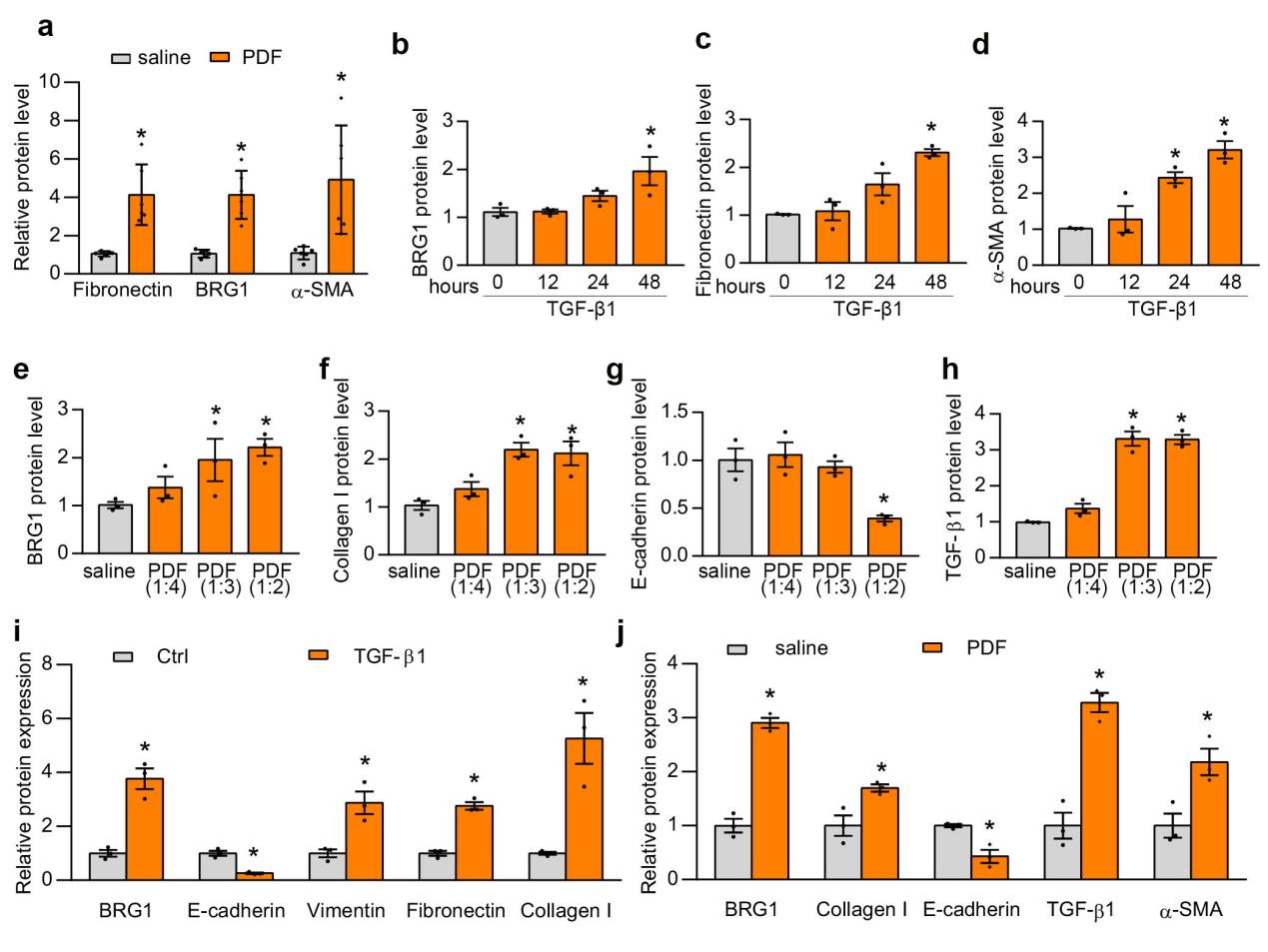


**Figure S1. BRG1 is increased in a mouse model of PD and in peritoneal mesothelial cells with TGF-β1 or PD fluid stimulation. (a)** Quantitative data of Western blot analyses show the visceral peritoneum expression of BRG1, Fibronectin, a-SMA. *P<0.05 versus saline mice.n=6 per group. **(b-d)** Quantitative data of Western blot analyses show the expression of BRG1**(b)**, Fibronectin**(c)**, a-SMA**(d)** in HMrSV5 cells challenged by TGF-β1 for 0, 12, 24 and 48 hours, respectively. *P<0.05 versus time zero. **(e-h)** Quantitative data of Western blot analyses show the expression of BRG1**(e)**, Collagen I**(f)**, E-cadherin**(g)** and TGF-β1**(h)** in HMrSV5 cells stimulated by PDF. *P<0.05 versus saline. **(i)** Quantitative data of Western blot analyses show the expression of BRG1, Collagen I, E-cadherin, Vimentin,and Fibronectin in RPMCs stimulated by TGF-β1. *P<0.05 versus control group. **(j)** Quantitative data of Western blot analyses show the expression of BRG1, Collagen I, E-cadherin, TGF-β1,and a-SMA in RPMCs stimulated by PDF. *P<0.05 versus saline.


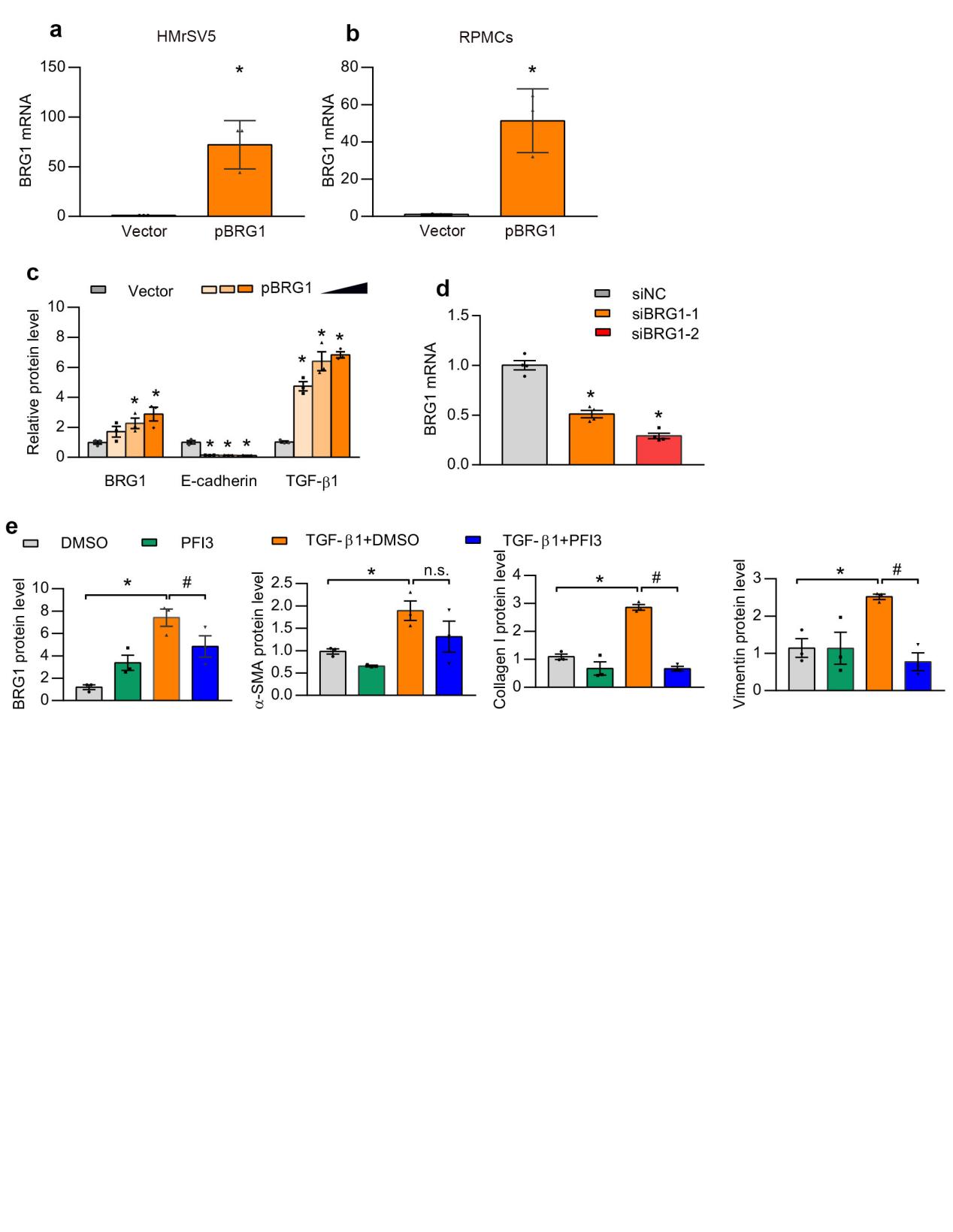


**Figure S2. BRG1 promotes fibrotic responses in vitro. (a)** Graphic presentation shows the mRNA expression of BRG1 in HMrSV5 cells in different groups as indicated. The mRNA level of BRG1 was detected by Quantitative real-time PCR .*P<0.05 versus Vector. **(b)**  Graphic presentation shows the mRNA expression of BRG1 in RPMCs in different groups as indicated.*P<0.05 versus Vector. **(c)** Quantitative data of Western blot analyses show the expression of BRG1, E-cadherin,TGF-β1. Triangle represents incremental plasmid dosage(2ug,3ug and 4ug/plate). *P<0.05 versus Vector. **(d)** Graphic presentation shows the mRNA expression of BRG1 in different groups as indicated in RPMCs.*P<0.05 versus Vector. (**e**) Quantitative data of Western blot analyses show the expression of BRG1, Vimentin, a-SMA, Collagen I in different groups are presented. *P<0.05 versus siNC,^#^P<0.05 versus siNC in the presence of TGF-β1.


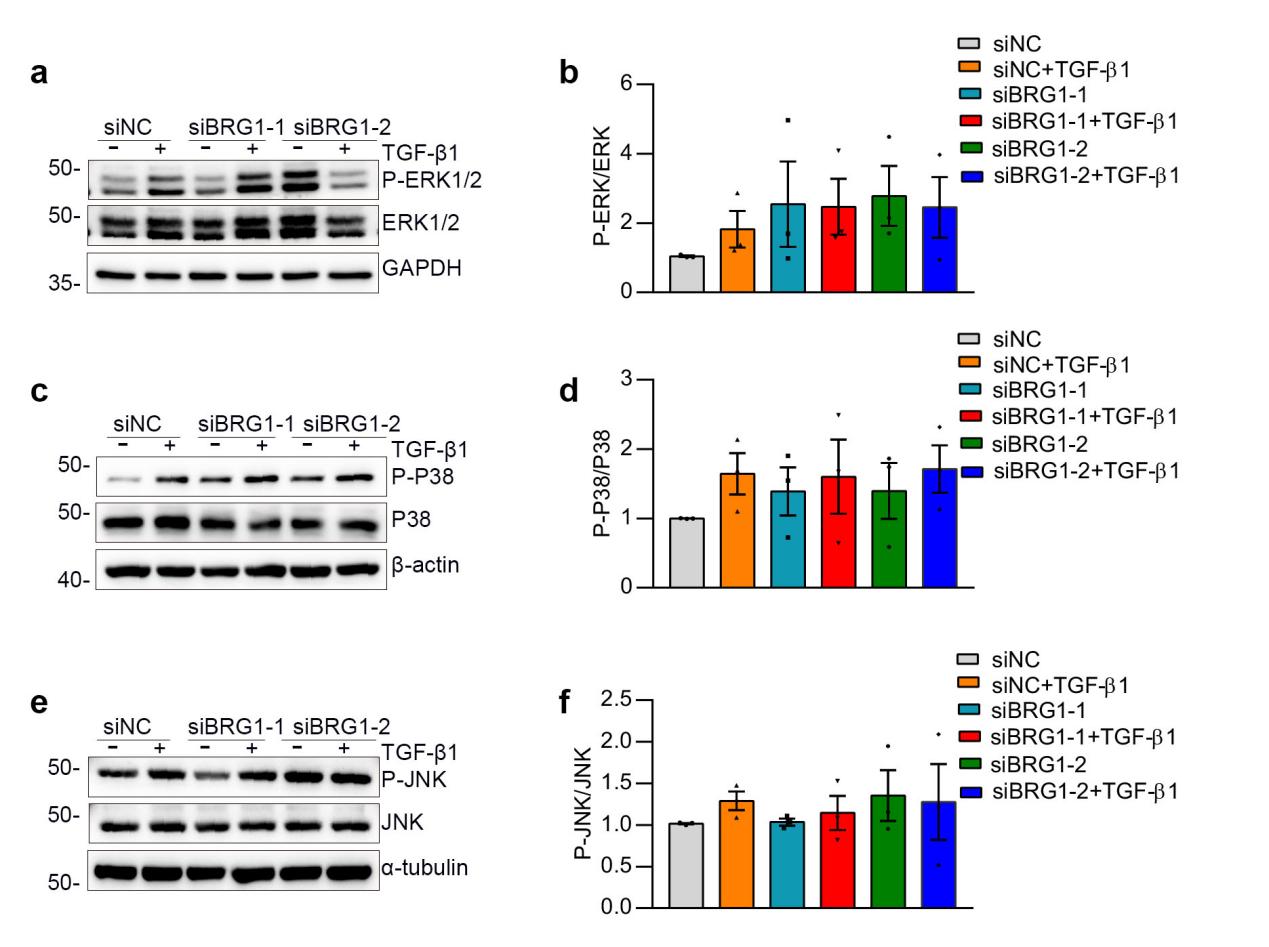


**Figure S3. BRG1 inhibition has no effect on phosphorylation of ERK1/2, P38 MAPK and JNK in vitro.(a-b)** Western blot analyses show that BRG1 inhibition by siRNA had no effect on phosphorylation of ERK1/2. Representative Western blot **(a)** and quantitative data **(b)** in different groups are presented. **(c-d)** Western blot analyses show that BRG1 inhibition had no effect on P38 phosphorylation. Representative Western blot **(c)** and quantitative data **(d)** in different groups are presented. **(e-f)** Western blot analyses show that BRG1 inhibition had no effect on phosphorylation of JNK. Representative Western blot **(e)** and quantitative data **(f)** in different groups are presented.


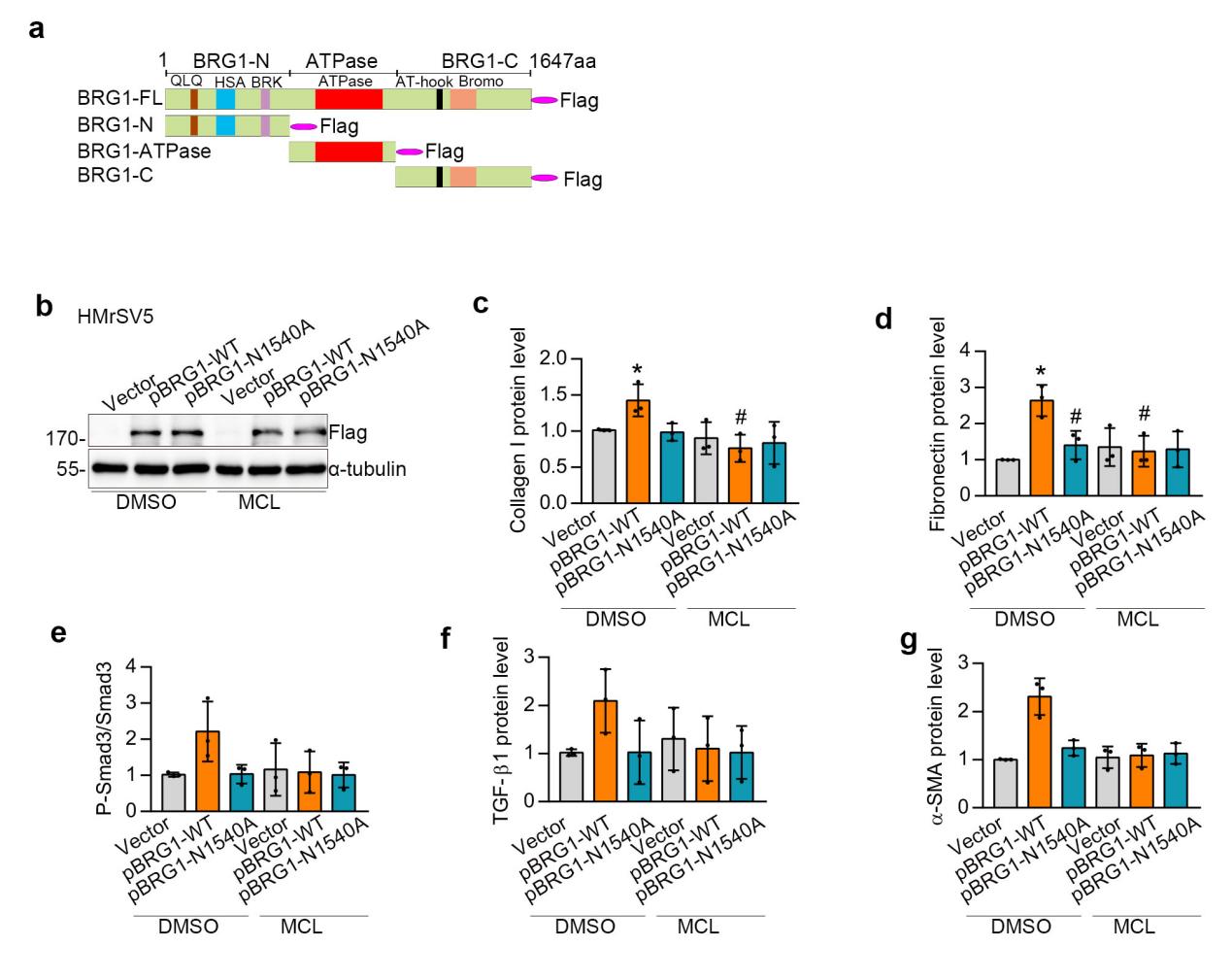


**Figure S4. The role of MCL on BRG1-induced fibrotic responses. (a)** Schematic representation of BRG1 mutants. BRG1 was divided into three regions: a ATPase domain,a N-terminal region, and a C-terminal region. Each mutant expression plasmids contains a C-terminal flag epitope tag. **(b)**Western blot analyses show that expression of Flag after BRG1 full length or BRG1 N1540A mutant transfection. **(c-g)** Quantitative data of Western blot analyses show the expression of Collagen I**(c)**, Fibronectin**(d)**, P-Smad3**(e)**, TGF-β1**(f)** and a-SMA**(g)** in different groups are presented. *P<0.05 versus Vector.#P<0.05 versus pBRG1.

**Table S1 The detail information of antibodies used in this study.**

| Name | Manufacturer | Product code | Host | Application |
| --- | --- | --- | --- | --- |
| anti-BRG1 | Abcam | ab110641 | Rabbit | WB,IHC,IP |
| anti-Collagen I | BOSTE | BA0325 | Rabbit | WB,IHC |
| anti-E-cadherin | BD Biosciences | 610181 | Mouse | WB,IF |
| anti-β-actin | EarthOx | E021020-01 | Mouse | WB |
| anti-TGF-β1 | Abcam | ab215715 | Rabbit | WB |
| anti-fibronetin | Abcam | ab2413 | Rabbit | WB,IHC,IF |
| anti-α-SMA | Abcam | ab124964 | Rabbit | WB,IF |
| anti-GAPDH | EarthOx | E021010-03 | Mouse | WB |
| anti-Vimentin | Santa Cruz Biotechnology | sc-373717 | Mouse | WB,IF |
| anti-α-tubulin | Beijing Ray | RM2007 | Mouse | WB |
| anti-P-Smad2 | Cell Signaling Technology | 18338 | Rabbit | WB,IF |
| anti-Smad2 | Cell Signaling Technology | 5339 | Rabbit | WB |
| P-Smad3 | Cell Signaling Technology | 9520 | Rabbit | WB,IF |
| anti-Smad3 | Cell Signaling Technology | 9523 | Rabbit | WB |
| anti-P-P38-MAPK | Cell Signaling Technology | 4511 | Rabbit | WB |
| anti-P38-MAPK | Cell Signaling Technology | 8690 | Rabbit | WB |
| anti-P-ERK1/2 | Cell Signaling Technology | 4370 | Rabbit | WB |
| anti-ERK1/2 | Cell Signaling Technology | 4695 | Rabbit | WB |
| anti-P-JNK | Cell Signaling Technology | 4671 | Rabbit | WB |
| anti-JNK | Cell Signaling Technology | 9252 | Rabbit | WB |
| anti-Histone H3 | Cell Signaling Technology | 4499 | Rabbit | WB |
| anti- acetyl-Histone H3 (Lys14) | Sigma-Aldrich | MABE351 | Mouse | WB |
| anti-FLAG | Sigma-Aldrich | F1804 | Mouse | WB |
| Dylight 488 anti-Mouse IgG | Abbkine | A23210 | Goat | IF |
| Dylight 594 anti-Mouse IgG | Abbkine | A23410 | Goat | IF |
| Dylight 488 anti-Rabbit IgG | Abbkine | A23220 | Goat | IF |
| Dylight 594 anti- Rabbit IgG | Abbkine | A23420 | Goat | IF |
| Rabbit IgG Control | Cell Signaling Technology | 2729 | Rabbit | IP |

Abbreviations: WB: western blot; IHC: immunohistochemistry; IF: immunofluorescence; IP: immunoprecipitation.

**Table S2. The sequence of siRNA.**

| RNAi | Target sequence |
| --- | --- |
| Rat-siBRG1-1 | GCGGCAAGTTCAATGTCTT |
| Rat-siBRG1-2 | CCGTGCAACAAACCATAAA |
| Homo-siBRG1-1 | CTCGGTCCGTCAAAGTGAA |

**Table S3. RT-qPCR primer sequences.**

| Gene | Forward(5’-3’) | Reverse(5’-3’) |
| --- | --- | --- |
| Rat-SMARCA4 | CCAGAATGCTCAGACCTTCAACCTC | TCCTCGCCTTCACTGTCATCCTC |
| Rat-GAPDH | GACATGCCGCCTGGAGAAAC | AGCCCAGGATGCCCTTTAGT |
| Homo-SMARCA4 | GACAAGGACGACGAGAGCAAGAAG | TGGTGAGGTTGGGTGGGTTAGG |
| Homo-β-actin | CCTGGCACCCAGCACAAT | GGGCCGGACTCGTCATAC |
